# Insights into the mechanism of the *PIK3CA* E545K activating mutation using MD simulations

**DOI:** 10.1038/s41598-018-27044-6

**Published:** 2018-10-19

**Authors:** Hari Leontiadou, Ioannis Galdadas, Christina Athanasiou, Zoe Cournia

**Affiliations:** Biomedical Research Foundation, Academy of Athens, 4 Soranou Ephessiou, 11527 Athens, Greece

## Abstract

Phosphoinositide 3-kinase alpha (PI3Kα) is involved in fundamental cellular processes including cell proliferation and differentiation and is frequently mutated in human malignancies. One of the most common mutations is E545K, which results in an amino acid substitution of opposite charge. It has been recently proposed that in this oncogenic charge-reversal mutation, the interactions between the protein catalytic and regulatory subunits are abrogated, resulting in loss of regulation and constitutive PI3Kα activity, which can lead to oncogenesis. To assess the mechanism of the PI3Kα E545K activating mutation, extensive Molecular Dynamics simulations were performed to examine conformational changes differing between the wild type (WT) and mutant proteins as they occur in microsecond simulations. In the E545K mutant PI3Kα, we observe a spontaneous detachment of the nSH2 PI3Kα domain (regulatory subunit, p85α) from the helical domain (catalytic subunit, p110α) causing significant loss of communication between the regulatory and catalytic subunits. We examine the allosteric network of the two proteins and show that a cluster of residues around the mutation is important for delivering communication signals between the catalytic and regulatory subunits. Our results demonstrate the dynamical and structural effects induced by the p110α E545K mutation in atomic level detail and indicate a possible mechanism for the loss of regulation that E545K confers on PI3Kα.

## Introduction

**Figure 1:**
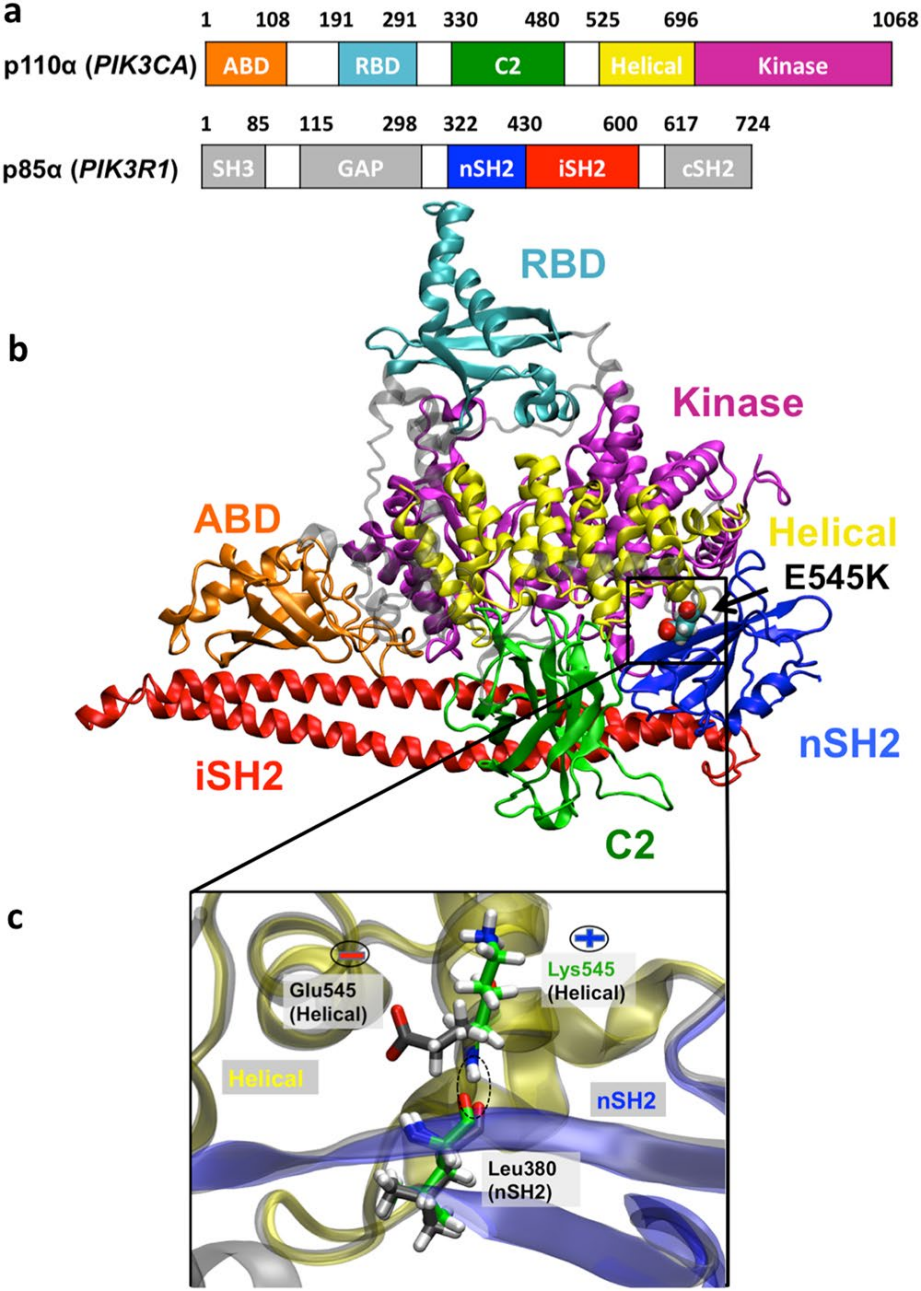
PI3Kα crystal structure and organization in functional domains. (**a**) Domain organization of the p110α (catalytic) and p85α (regulatory) subunits. (**b**) Structural overview and schematic representation of the domain organization of the PI3Kα p110α/p85α heterodimer indicating the position of the E545K mutation site. The nSH2 domain occupies a critical position interacting with the helical, C2, and kinase domains of the catalytic subunit. (**c**) Magnification of the nSH2 (p85α)-helical (p110α) interface at the E545K mutation site. Residues E/K545 (helical) and L380 (nSH2) are shown in stick representation (carbon atoms of the mutant are colored in green and of the wild type (WT) protein in grey). Superposition of the WT and E545K PI3Kα mutant (the nSH2 domain is colored in blue and the helical domain in yellow) illustrates the different orientations that E545(−) or K545(+) adopt in the initial protein conformations prior to the simulations.

Phosphoinositide 3-kinase alpha (PI3Kα) is a lipid kinase that plays a crucial role in cell processes involving growth and survival^1^. PI3Kα is comprised of a catalytic subunit, p110α, encoded by the *PIK3CA* gene, and a regulatory subunit, p85α, encoded by the *PIK3R1* gene (Fig. 1). Somatic missense mutations of p110α have been identified as the most common mutations associated with a variety of human cancer types^2–8^. The two most common cancer-linked mutations are H1047R and E545K, located in the kinase and helical domains of p110α, respectively^6,9^. These hotspot mutations transform cells *in vitro*^10^ and enhance tumorigenicity in transgenic mouse models^11^. Currently, several PI3Kα inhibitors are under development and in clinical trials^12^. Alone or in combination with other drugs, PI3Kα inhibitors prevent tumor growth particularly in estrogen receptor alpha (ERα)-positive breast cancers, but depending on the type of the PI3Kα mutation different therapeutic regimes must be followed^13^.

Structural and functional studies of the PI3Kα wild type (WT) and hotspot mutants are of paramount importance for understanding the effect of oncogenic mutations on the constitutive lipid kinase activity of *PIK3CA* (p110α subunit) and consequently for the discovery of mutant-specific inhibitors. The crystal structure elucidation of PI3Kα WT^14–17^ and H1047R mutant^18^ has enabled the mechanistic understanding of PI3Kα overactivation by the H1047R mutant. By using a combination of Molecular Dynamics (MD) simulations and experiments, it was shown that the H1047R mutation, which is located in the kinase domain of PI3Kα, abolishes the auto-inhibitory role of the C-terminal tail of the protein, enhances protein-membrane binding, and alters the conformation of H917, a critical residue for ATP hydrolysis^19,20^. Although a crystal structure bearing the PI3Kα E545K mutation, which is located in the helical domain of PI3Kα, is not yet available, several studies suggest that this mutation leads to elevated kinase activity by an entirely different mechanism than H1047R. Moreover, it has been shown in biochemical experiments that when both mutations are introduced they act independently and synergistically, indicating that the molecular mechanisms for the mutation-induced gain of function in helical and kinase domain mutations are complementary and reciprocal^21^. Helical domain mutants (such as E545K) lead to activation by abolishing the inhibitory contact of p85α that regulates PI3Kα activation (such mutants are p85α independent), however, they still need to interact with RAS to reach the membrane. On the other hand, while kinase domain mutants (such as H1047R) are active in RAS absence, they require the interaction with the regulatory subunit p85α^21^.

Early studies have indicated that E545K acts by altering the p110α-p85α interface^22,23^. In a low activity state, the nSH2 and iSH2 domains of the regulatory subunit p85α stabilize p110α and inhibit its enzymatic activity. In one of these inhibitory contacts, the nSH2 domain of p85α interacts strongly with the helical domain of p110α and stabilizes the inactive state of PI3Kα. This contact is physiologically released by phosphotyrosine (pY)-containing peptides, which bind at the nSH2-helical interface and activate the enzyme (pY-activated state of the WT)^24,25^. The E545K charge reversal mutation in the helical domain of p110α is located exactly at the position where pY-peptides bind nSH2. Later studies also suggest that the E545K mutant increases lipid kinase activity by disrupting the nSH2-helical interface, mimicking the pY-activated state of the protein^21,26–28^. This mechanism has been further supported by biochemical studies investigating the kinetics of PI3Kα activation for the WT and oncogenic mutant (E545K, H1047R) proteins^27,29^. Both mutants show a 2-fold increase in lipid kinase activity compared to the activated WT^29^. However, contrary to H1047R, the E545K mutant cannot be further activated in the presence of growth factor stimulation (pY-peptides), suggesting that this mutant activates p110α through the same mechanism as the binding of pY-peptides^27^. Nevertheless, so far, there is no crystal structure of the mutant E545K available to provide structural evidence for this mechanism.

A recent hydrogen/deuterium exchange mass spectrometry (HDX-MS) study gave the first insight into the structural mechanism of activation of PI3Kα caused by the E545K mutation^30^. Burke *et al*. showed that the E545K helical mutation enhances three out of the four distinct events that occur during the physiological transition of the WT PI3Kα from an inactive to an active state. These three events require breaking of the nSH2-helical interface, disruption of the iSH2-C2 interface, and movement of the ABD domain relative to the rest of the catalytic subunit^30^. Although the experiments capture the dynamic nature of the enzyme activation, the order with which these events are taking place remains unclear.

MD simulations have been successfully used for the identification of intermediate structures during the activation pathway of various kinases^19,31^. Conventional MD^32–34^ or biased, enhanced sampling methodologies^35–37^ have been extensively used to study the mechanism of constitutive activation of various protein kinases as well as the mechanism of action of anti-cancer drugs^35,37,38^. A recent MD study of oncogenic mutations of PI3Kα suggests that all tumor-associated mutants weaken p110α-p85α interactions by increasing positional fluctuations of nSH2 (p85α) domain^39^. However, the limited sampling of these simulations could not allow the observation of significant conformational changes. Another recent study of E545K and E542K mutants of PI3Kα, involving docking of catalytic (p110α) and regulatory (p85α) subunits and subsequent MD simulations, indicates fewer interactions of p110α-p85α in the mutants compared to the native structure^40^. In this study, a connection between nSH2 (p85α) flexibility and helical mutations (E545K, E542K) was observed, however, it remains unclear how this effect could eventually lead to nSH2 detachment and allosteric regulation of the enzyme activity, perhaps due to the approximation of docking the two subunits and not using the crystal structure in the simulations.

In the present study, we performed microsecond-long simulations of the full-length WT and E545K p110α/niSH2 heterodimer to investigate the structural and dynamical changes induced by the mutation through unbiased MD simulations. For the first time, we have been able to follow in atomic-level detail the unforced detachment of nSH2 (p85α) from the helical (p110α) domain in the PI3Kα mutant form, monitoring the series of events leading to this disruption. The molecular mechanism that emerges from our study could explain how the nSH2 domain detaches gradually from the helical one, resulting in loss of this inhibitory contact that can eventually lead to overactivation of the enzyme. We also investigated the allosteric network connecting key residues in the WT and mutant proteins and show that a cluster of residues around 545 is critical for delivering communication signals between the catalytic and regulatory subunits. Our simulations are in agreement with HDX experimental data and allow us to provide atomic-detail insights into the mechanism of the *PIK3CA* E545K activating mutation by monitoring structural and dynamical elements of the WT and mutant proteins.

## Results

### Spontaneous detachment of the nSH2 (p85α) domain from the p110α subunit

#### Disruption of nSH2-helical interface in the E545K mutant

**Figure 2:**
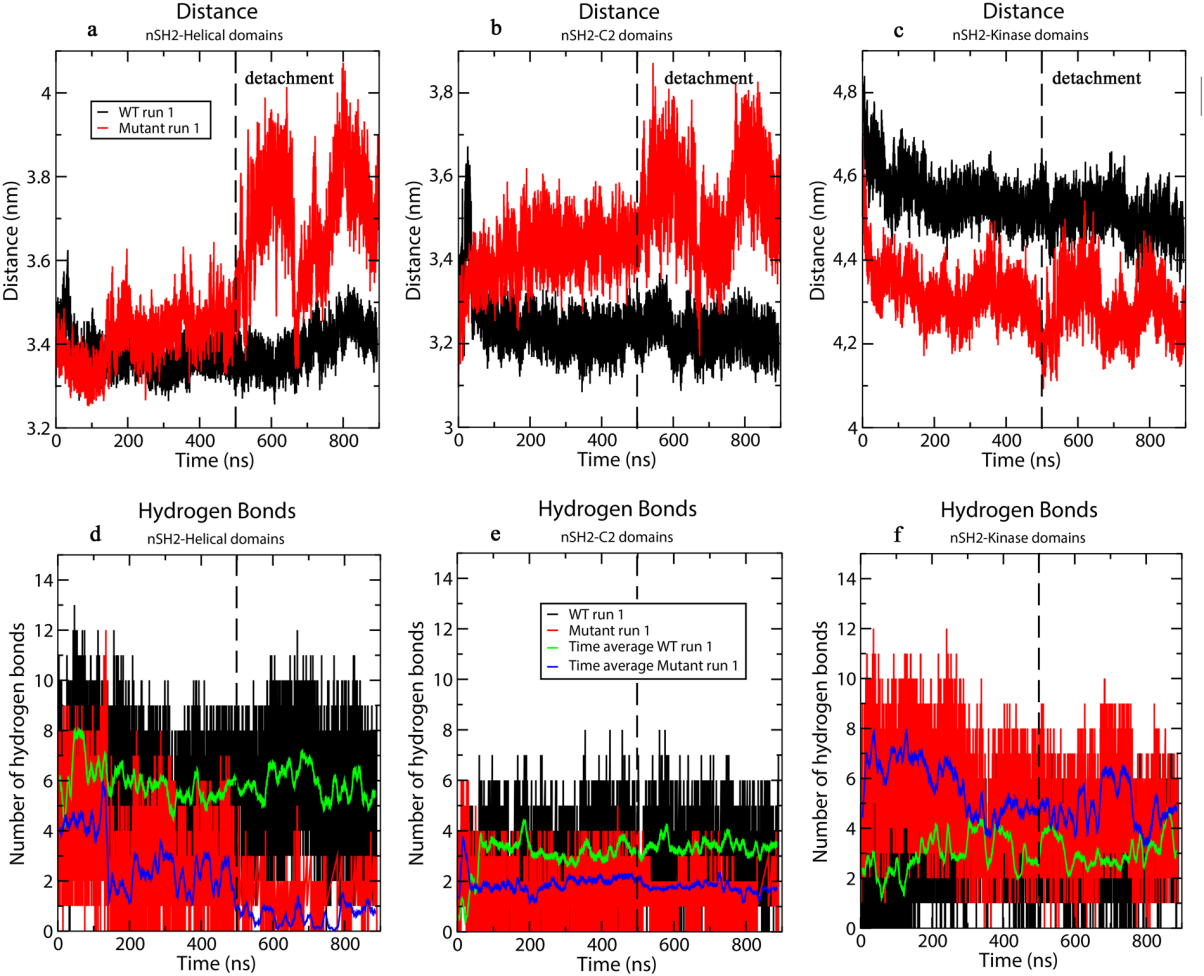
Time series of the distance between the centers of mass of (**a**) nSH2-helical, (**b**) nSH2-C2, and (**c**) nSH2-kinase domains. The dotted line indicates the time, when nSH2 begins to detach from the helical and C2 domains. The time dependence of the total number of hydrogen bonds between (**d**) nSH2-helical, (**e**) nSH2-C2, and (**f**) nSH2-kinase domains is also presented. The black line represents results from the WT run 1 simulation and the red line represents results from the Mutant run 1 simulation (see Table 1 for simulation details).

Calculation of the Root Mean Square Deviation (RMSD) with respect to the initial structure shows that a significant conformational change takes place in one of the PI3Kα E545K mutant simulations (Mutant run 1, Supplementary Fig. S1). Also, as seen in Supplementary Fig. S2, the Root Mean Square Fluctuation (RMSF) of the nSH2 domain is significantly increased in the mutant compared to the WT. A detailed analysis of the conformational change shows that it is attributed mostly to the movement of the nSH2 domain (p85α) relatively to the helical one (p110α) (Fig. 2). Initially, the nSH2-helical domains are in close contact forming several hydrogen bonds (closed-state) as in the WT crystal structure (a detailed description of the polar network interactions is given at the end of this section). Destabilization of this interaction network at the nSH2-helical interface in the mutant protein, triggers a significant conformational change. nSH2 gradually moves away and detaches from the helical domain in accordance with previous HDX experiments^30^ and structural studies^18^. The movement of nSH2 relative to the helical domain is clearly seen in Fig. 2a, which shows the distance between the center of mass of the two domains increasing upon the detachment. Notice, that after the detachment (t = 500 ns, dotted lines in Fig. 2) nSH2 approaches the helical domain for a short period (transient attachment, t = 670 ns), but then again moves away (t = 800–900 ns, Fig. 2a). A step-wise character of the disruption is revealed by examining the time series of the total number of hydrogen bonds formed between nSH2-helical (Fig. 2d). At t = 200 ns, the total number of hydrogen bonds between nSH2-helical decreases from 4 to 2 (Step 1). The decrease in the total number of hydrogen bonds results in increasing fluctuations of the distance between the centers of mass of nSH2-helical domains (Fig. 2a). At t = 500 ns (Step 2) an almost complete nSH2-helical hydrogen bond loss takes place as can be seen in Fig. 2d. The nSH2-helical hydrogen bond network disruption leads to the actual detachment, where a 5 Å increase in the nSH2-helical center-of-mass distance is observed (open-state). A transient re-attachment of nSH2 occurs at around 670 ns due to the formation of a hydrogen bond between the nSH2-helical domains (Step 3), which, however, is lost at around 800 ns and the nSH2 detaches from the helical domain at the end of the simulation run (Step 4). The total number of hydrogen bonds between nSH2-helical domains is consistently lower in all mutant simulations with respect to the WT (Supplementary Figure S3). On the contrary, the nSH2-helical distance and the total number of hydrogen bonds between the two domains (Fig. 2a, d and Supplementary Figure S3) in the WT protein remain constant indicating a stable nSH2-helical contact. For a detailed explanation of the sequence of events observed upon detachment and the role of the charge reversal, see the Supplementary Information. A video with the trajectory of the detaching nSH2 domain from the helical domain can be seen in Supplementary Video S1.

**Table 1:** List of the simulation time for all studied systems.

Simulated System	Simulation Time (ns)
WT run 1	900
WT replica 1	800
Mutant run 1 (K379 unprotonated)	900
Mutant replica 1 (K379 unprotonated)	900
Mutant replica 2 (K379 unprotonated)	900
Mutant replica 3 (K379 unprotonated)	900
Mutant replica 4 (K379 protonated)	1800
Mutant replica 5 (K379 protonated)	1800
Mutant replica 6 (K379 protonated)	1800
Mutant replica 7 (K379 protonated)	1800

**Figure 3:**
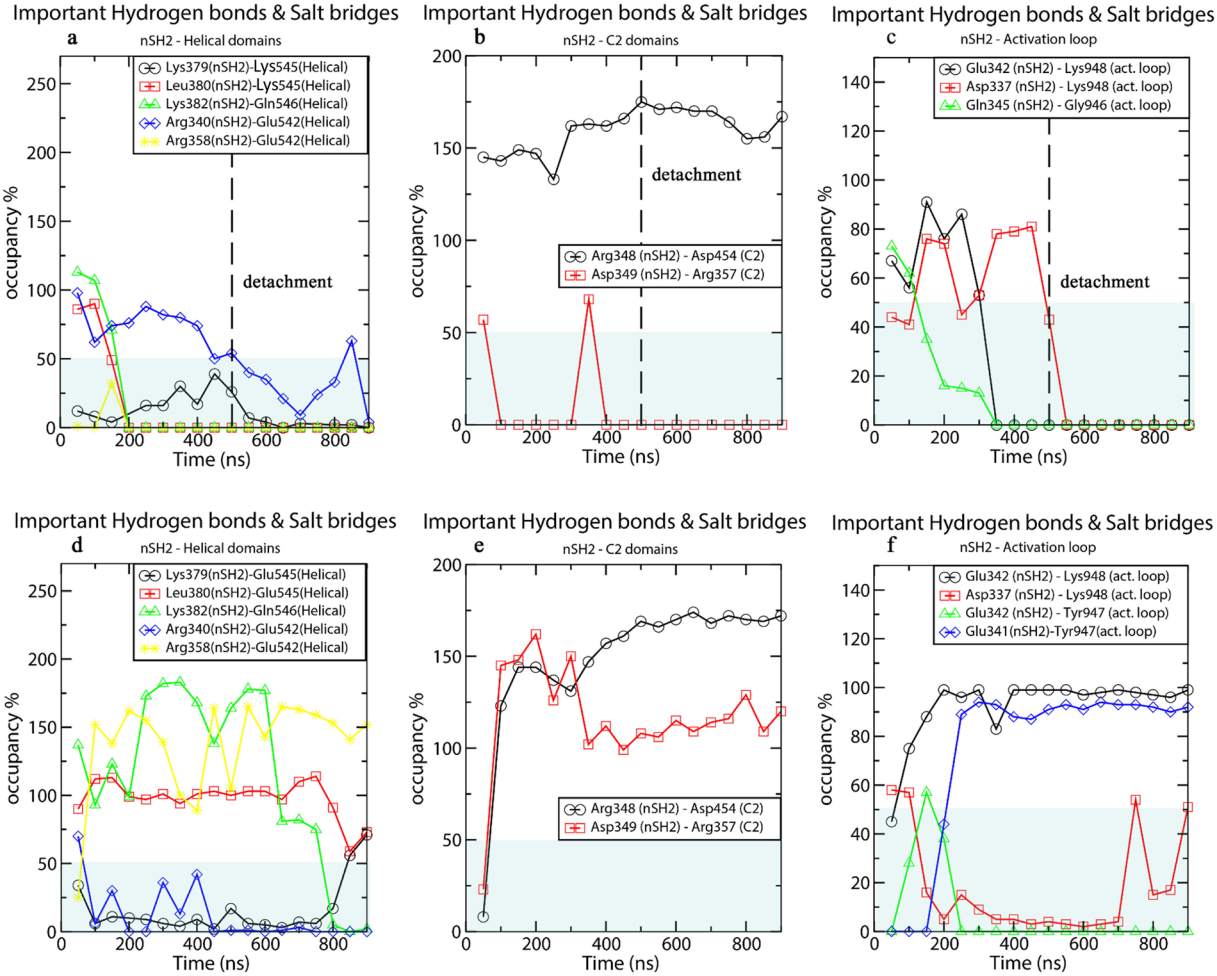
Time average (every 50 ns) of hydrogen bonds in the vicinity of E545K (the grey box marks occupancy <50%) between (**a**) nSH2-helical and (**b**) nSH2-C2 domains (**c**) nSH2-activation loop for the Mutant run 1 simulation (the black dotted line indicates the time of the detachment of nSH2 from the helical domain), and (**d**, **e**, **f**) for the respective interfaces in the WT run 1 simulation, where no detachment is observed.

To better understand the hydrogen bond disruption during the nSH2-helical detachment, we have calculated the time series of nSH2-helical hydrogen bonds, which are discussed below. The percentage of occupancy of inter-domain hydrogen bonds in the vicinity of the E545K mutation for all simulated systems can be found in Supplementary Tables S1-S3. Here, we do not focus on the total number of hydrogen bonds (that could involve the formation of transient hydrogen bonds between residues at the nSH2-helical interface), but rather on the formation and disruption of persistent hydrogen bonds between specific interfacial residues. Breaking of these hydrogen bonds could possibly trigger the observed conformational change. As seen in Fig. 3a and Supplementary Table S1, the L380(nSH2)-K545(helical) and K382(nSH2)-Q546(helical) hydrogen bonds in the Mutant run 1 simulation, are the first to be disrupted as a result of the weakening of the bond K379-545K, which ensues after the charge-reversal mutation (Supplementary Table S3) (Step 1). The only remaining hydrogen bond in the vicinity of K545 is R340(nSH2)-E542(helical), which keeps the two domains in close contact. The disruption of this last strong inter-domain hydrogen bond interaction (Step 2) initiates the movement of nSH2 away from the helical domain. The two domains associate transiently again due to the formation of the critical hydrogen bond R340(nSH2)-E542(helical) (Fig. 3a) (Step 3), but then the loss of this hydrogen bond leads again to the dissociation of the two domains (Step 4). In a previous experimental study, it was shown that the p85ni-R340E mutant did not inhibit the WT p110α, and it was suggested that R340 is involved in an inhibitory contact with the catalytic subunit^25^.

Apparently, E542 acts as the last residue to preserve the nSH2-helical inhibitory contact. It should be noted that a persistent interaction of E542 (R358(nSH2)-E542(helical)) in the WT, is almost completely absent in the mutant E545K simulation (Fig. 3a,d and Supplementary Tables S2 and S3). In fact, we show in our allosteric analysis (see the “Dynamical Network Analysis” Section) that the communication between R358(nSH2)-E542(helical) is weakened as an effect of the mutation E545K. The importance of the E542(helical) residue in preserving the inhibitory role of nSH2 is well known. Biochemical analysis of a double E545K-E542K mutant has revealed a simple additive behaviour in the enzyme overactivation, suggesting that the two helical mutations act through the same mechanism, which involves the release of the nSH2-helical inhibitory contact^21^.

#### Effect of E545K on the nSH2-C2 and nSH2-kinase interfaces

The detachment of nSH2 from the helical domain in the mutant protein also alters the interactions of nSH2 with the neighboring C2 and kinase domains of the catalytic p110α subunit. As seen in Fig. 2b, the center of mass distance between nSH2 and C2 is initially 3.3 nm, but after the detachment it oscillates between 3.3 ± 0.1 and 3.8 ± 0.1 nm. In contrast, in the case of the WT run 1 simulation, the center of mass distance between nSH2 and C2 remains stable at 3.2 ± 0.1 nm. In all mutant simulations (Table 1 and Supplementary Fig. S3), the nSH2 domain approaches the kinase domain starting from 4.8 ± 0.1 nm and reaching to a mean value of 4.4 ± 0.1 nm. In the WT simulations, the nSH2 – kinase center of mass distance remains stable at 4.3 ± 0.1 nm (Fig. 2). Despite the fact that the relative positioning of nSH2 changes, the total number of hydrogen bonds between nSH2-C2 and nSH2-kinase domains is constant throughout the simulation (Fig. 2e, f) and is not affected by the detachment. Overall, the total number of hydrogen bonds between nSH2-C2 and nSH2-kinase domains ranges between 2–4 and 4–6, respectively, for all simulated systems (Fig. 2, Supplementary Fig. S3 and Supplementary Table S3). Therefore, even though nSH2 has lost contact with the helical domain of the p110α catalytic subunit during detachment, it maintains essential polar contacts with the other two neighboring domains (C2, kinase) that presumably keep it from moving far away from the complex.

**Figure 4:**
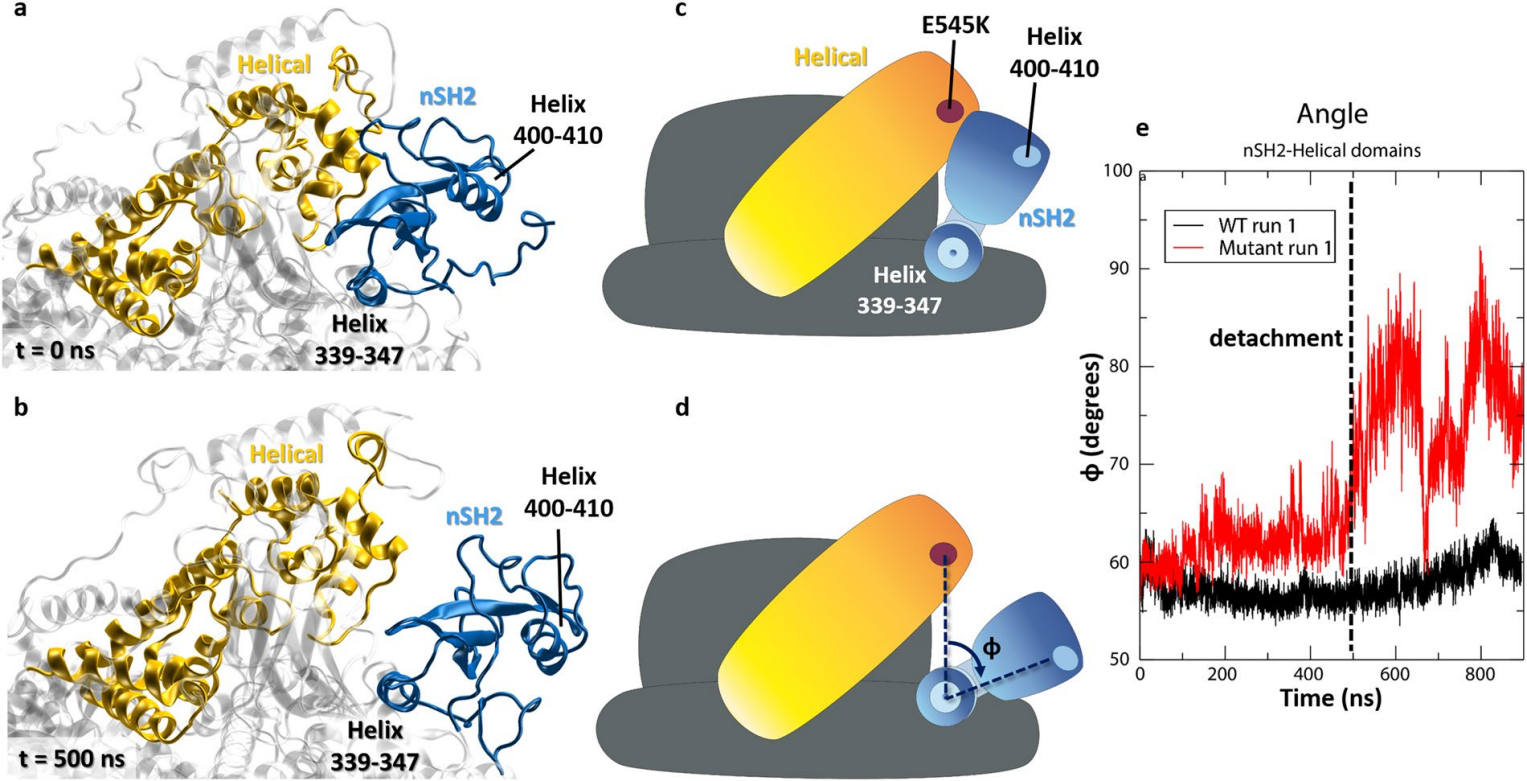
Rotational detachment of the nSH2 from the helical domain, around helix 339−347 (nSH2 domain). (**a**) Initial (t = 0 ns) conformation of nSH2 and helical domains in the Mutant run 1 simulation. (**b**) Conformation of the two domains upon the first detachment (t = 500 ns) in the Mutant run 1 simulation. The nSH2 domain rotates around helix 339−347, which serves as a hinge, and detaches from the helical domain. (**c**), (**d**) Graphical illustrations of the (**a**) and (**b**) conformations. (**e**) Time series of the angle φ (depicted in (**d**)) for the WT and Mutant run 1 simulations.

nSH2 contacts the kinase domain with its 339–347 helix, which interacts with helix α10 of the C-lobe of the kinase domain. We observe that during the detachment, the 339–347 helix serves as a hinge around which nSH2 rotates and detaches from the helical domain (Fig. 4). As seen in Fig. 4e, the angle φ (shown in Fig. 4d) that is formed between the center of mass of residue E545K, the center of mass of helix 339–347, and that of helix 400–410 (which is located in the solvent exposed part of the nSH2 domain), ranges from 60°, at the beginning of the simulation (Fig. 4a, c), to 90° upon the detachment (Fig. 4b, d). According to HDX-MS data, “the one area in the nSH2 that shows decreased HDX in p110α compared to p110β and p110δ is the helix A (residues 339–347). This may explain why the nSH2 more strongly inhibits the p110α subunit compared to p110β, and p110δ. Although there is no crystal structure of the p110β or p110δ interacting with the nSH2, the HDX-MS suggests that there are conformational differences with respect to p110α that result in more protection of the nSH2, likely due to it making more extensive contacts in the p110α/p85 complex”^41^. Indeed, our simulations show that this interaction persists until the end of our simulation, and we may hypothesize that the detachment described in the current work constitutes the first step for the complete dissociation of nSH2 from its neighboring counterparts, which will lead to constitutive relief of inhibition.

### Loss of communication between functional domains

It is well known that kinases have a dynamic infrastructure that can be disrupted by single point mutations, resulting in allosteric regulation of enzyme activity^42–45^ even in the absence of large conformational changes. In our case, where a significant conformational change takes place, we expect to see differences in the communication between domains of PI3Kα in the WT and E545K mutant proteins, indicating a possible mechanism for allosteric regulation of the enzyme activity controlled by E545K. Comparison of the RMSF of the E545K mutant simulation, where nSH2 detaches (open-state), with the WT (closed-state) shows that there are specific regions in functional domains that are more flexible in the mutant open-state. As seen in Supplementary Fig. S4, residues in certain regions of nSH2, helical, and iSH2 domains that are in proximity to the disrupted, open-state nSH2-helical interface show enhanced flexibility that is increased in the order of RMSF = 0.1–0.2 nm compared to the closed-state (Supplementary Fig. S5 and Table S4). The locations of the increased flexibility regions of the open-state protein are in agreement with HDX experiments^30^ (Supplementary Fig. S4). Specifically, increased fluctuations are observed for residues 374–424 that constitute a β-sheet and an α-helical part of nSH2 that is in close contact with the E545K mutation site and for residues 541–553 that include the mutation site on the helical domain (Supplementary Fig. S4). Furthermore, residues 442–452 (iSH2) of the regulatory subunit are also more flexible in the mutant open state compared to the rest of the simulation runs that are sampling the closed state (Supplementary Fig. S5 and Table S4). This flexibility could be expected as residues 442–452 of iSH2 are connected by chain continuity with the detached nSH2 domain. There are two additional regions, residues 374–380 of the C2 domain and residues 784–794 of the kinase domain that show increased flexibility in the mutant open-state. However, these specific regions are implicated neither in inter-domain nor in functionally important interactions.

#### Maps of distance fluctuations

**Figure 5:**
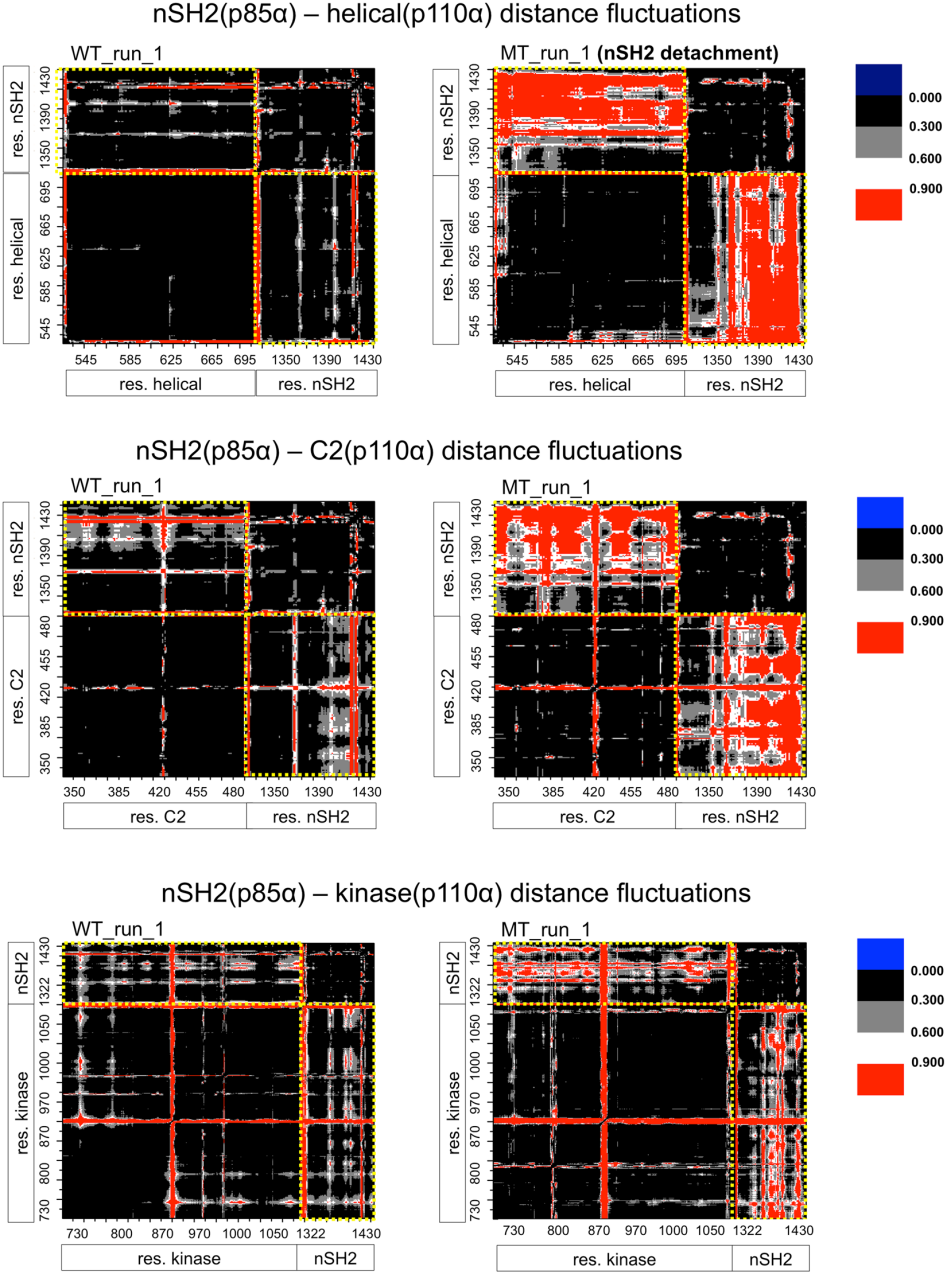
Maps of distance fluctuations (in Å^2^) for nSH2-helical, nSH2-C2, and nSH2-kinase domains calculated from the WT (closed-state) and mutant (open-state) simulation runs. Black areas designate the most rigid parts of the protein, while red areas indicate the strongest conformational variability. Yellow dotted lines include distance fluctuations between residues of the same domain, giving an indication of the inter-domain interactions.

In order to assess the effect of the E545K mutation on the plasticity and coordination between domains, we have calculated the distance fluctuation (DF) maps for the Cα atoms of the nSH2-helical, nSH2-C2 and nSH2-kinase domains^46^ (see Methods). The results are presented in Fig. 5 for the WT (closed-state) and the mutant (open-state) proteins. Values of distance fluctuations that are lower than 0.3 Å^2^ and correspond to pairs of atoms of nSH2-helical, nSH2-C2, and nSH2-kinase domains, indicate rigid body-like motions that could propagate strong allosteric communication between these domains. Therefore, as seen in the Mutant run 1 simulation (open-state, Fig. 5), the detachment of nSH2 results in significant distance fluctuations, weakening the communication between nSH2-helical and nSH2-C2 domains. Interestingly, although nSH2 seems to physically approach the kinase domain (Fig. 2c) the communication between the two is also distorted with respect to the WT (Fig. 5). The conformational plasticity has been also calculated for mutant replica runs (Table 1), however, it does not show a significant change compared to the WT protein (Supplementary Fig. S6).

#### Dynamical network analysis

In allosteric communication, a signal is propagated over a long distance through the protein structure from one site to another. It has been argued that allosteric communication in proteins relies upon networks of collective, rigid body motions and residue-residue contact motions^47–49^. To understand the role of residue E545K in allosteric regulation within PI3Kα, we have constructed network models obtained from our MD simulations. For our analyses, we used the Dynamical Network Analysis method as implemented in the program VMD^50,51^ (see Methods section and the SI for a detailed explanation). Based on this model, allosteric signals are dependent on positional correlations of protein residues, and correlated motion is used to generate a weight of the signal transmitted through two residues. Apart from identifying allosteric pathways, the method also applies hierarchical clustering using the Girvan-Newman algorithm to cluster residues whose motion is highly correlated in so-called “communities” that are highly intra-connected but loosely inter-connected (see SI for more information)^52^. The communication between different communities passes through specific residue interactions that form critical edges.

**Figure 6:**
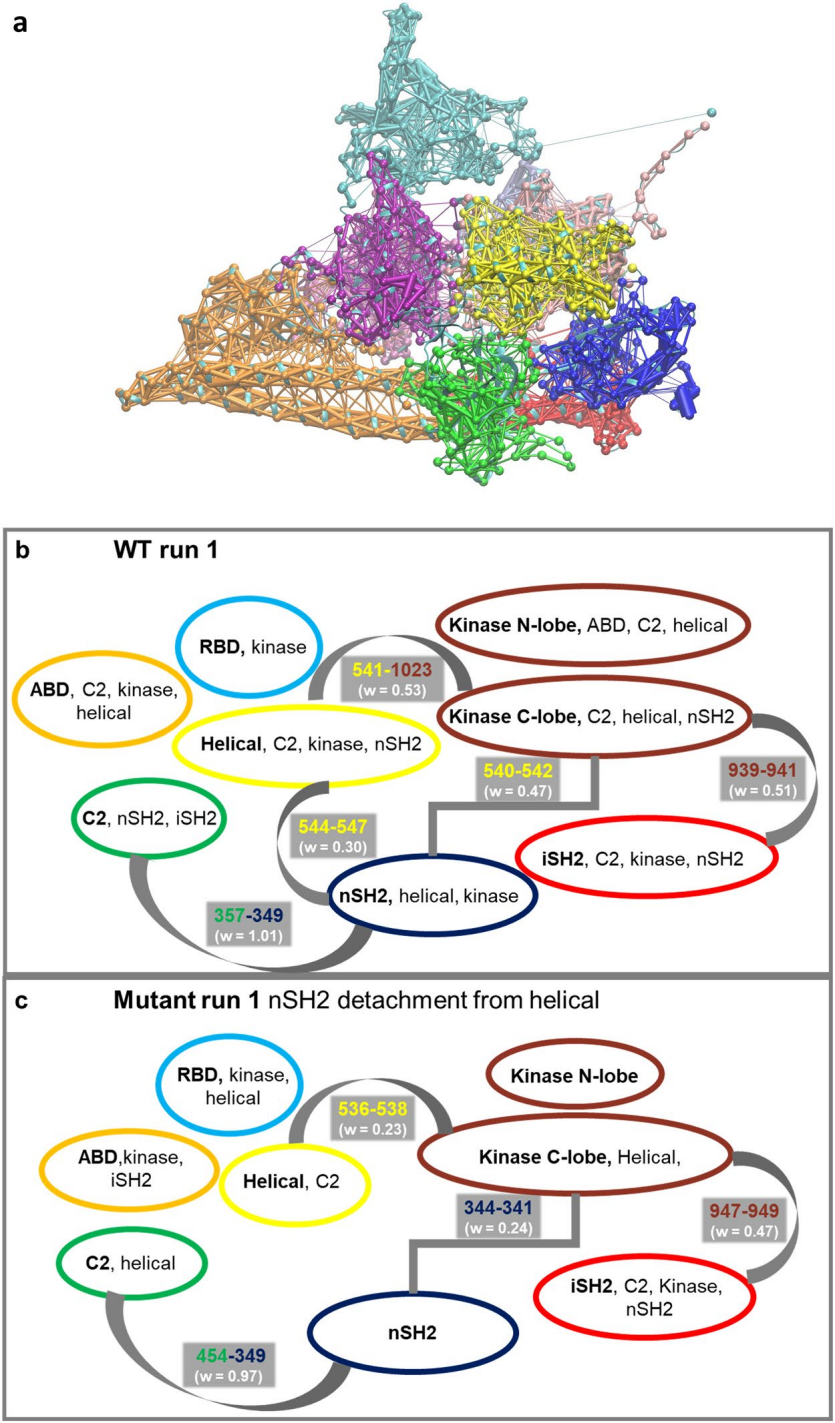
(**a**) Based on the dynamical network analysis a partitioning of the system can be performed, in which each node belongs to a specific community. Nodes of a community have much stronger connections within that community than to nodes of other communities. In this representation, we show as an example the PI3Kα WT partitioning of the residues in communities, based on their motional correlation. Note that, the community analysis largely reflects the organization in domains that has been proposed for PI3Kα, which is also schematically depicted in Fig. 1. Orange includes the ABD domain (1–108) and most of the iSH2 (471–560), cyan represents the RBD domain (190–300), red represents the remainder of the iSH2 domain (429–469 and 562–598), C2 is depicted in green (335–478), nSH2 is depicted in blue (322–428), the helical domain is shown in yellow (526–643), the N-lobe of the kinase domain is shown in magenta (699–808), and pink represents the C-lobe of the kinase domain (809–1067). (**b)**, (**c**) Clustering of communities based on the dynamical network analysis of PI3Kα WT (run 1) and E545K (Mutant run 1, open-state) proteins. Each community is represented by a ellipse including the main domain in bold and parts of other secondary domains all in the same community. Critical nodes connecting communities are shown with thick grey linestogether with the edge weights (in white letters). Notice, that the color coding of the ellipses and numbers of critical nodes is kept the same as in Fig. 1, representing the dominant domain in each community (ABD-orange, RBD-cyan, C2-green, helical-yellow, kinase-mauve, nSH2-blue and iSH2-red). The critical node 544-547 is lost in the open state (Mutant run 1).

A detailed analysis of the inter-domain communication network has been performed for the WT (closed-state) and mutant (open-state) using the dynamical network analysis method (see Methods section). As can be seen in Fig. 6a, the clustering of residues in “communities” largely reflects the organization of the functional domains that has been proposed for PI3Kα, which is also schematically depicted in Fig. 1. While the PI3Kα domains are very well characterized by the communities calculated from the network analysis, some residues act at the interface between domains and propagate their motion within the allosteric network of the protein, and thus, depending on their positional correlation they may be categorized in an adjacent PI3Kα domain (Fig. 6b, c). For the WT protein, the majority of the paths connecting residues between the nSH2 and helical domains pass through the edge defined by T544(helical community) and E547(nSH2 community) residues (Fig. 6b). Moreover, the nSH2 domain is additionally connected to the helical domain, through residues L540, P539, which belong to the helical community, and E542, R537, which belong to the nSH2 community, according to the network analysis. Clearly, the residues around the hotspot mutations E542/E545 play a critical role in the communication of the helical domain with the nSH2 domain (for more details about the edges between nSH2 and helical domains, the reader is referred to Supplementary Table S5). However, this communication is completely lost upon the detachment of nSH2 from the helical (Fig. 6c).

**Figure 7:**
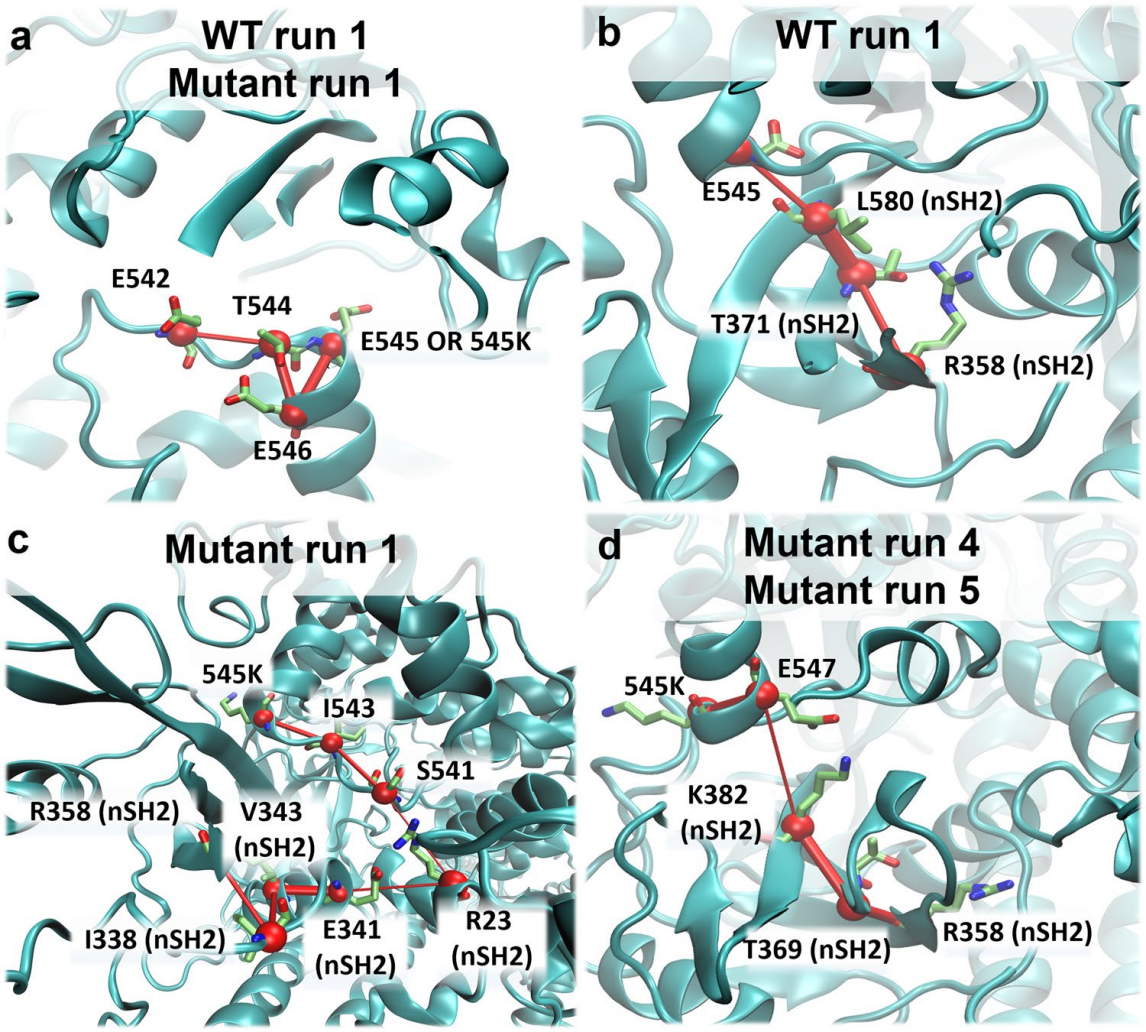
Communication distortion between residues 545K and R358. (**a**) Path of communication between E545 or 545K and E542 in WT and Mutant run 1 simulations. (**b**) Path of communication between E545 and R358 (nSH2) in WT run 1 simulation. (**c**) Elongated path of communication between 545K and R358 (nSH2) in Mutant run 1 simulation. (**d**) Elongated path of communication between 545K and R358 (nSH2) in Mutant run 4 and 5 (Table 1).

One of the key interactions between nSH2 and helical domains that is almost immediately lost in the majority of the E545K mutant simulations, is the R358-E542 salt bridge (Fig. 3a and Supplementary Table S3). Our network model indicates that the communication between 545K and E542 is preserved, while the communication between 545K and R358 is significantly reduced with respect to the WT (Fig. 7). The optimal path of communication between 545K and R358 is substantially longer compared to E545 – R358 (Fig. 7b-d). For the R358 – E542 salt bridge, the position of R358 is maintained close to E542 through a motional correlation with E545 (Fig. 7b), but when E545 is mutated to 545K, the weakening of the communication between E545 and R358 (Fig. 7c, d) leads to the breaking of the R358-E542 salt bridge.

The communication between nSH2-C2 domains is also significantly distorted in the mutant open-state. Although upon the nSH2-helical detachment the nSH2-C2 interface is largely affected, based on the maps of distance fluctuations (Fig. 5), one critical edge involving residue D349 nSH2 domain remains (Fig. 6), which may preserve the communication between the two domains. Another domain of the regulatory subunit, iSH2, directly interacts and possibly controls the kinase, C2, and ABD domains of the catalytic subunit. However, analysis of the communication network between these domains has shown that they are not significantly affected by the conformational change taking place at the helical-nSH2 interface. Interestingly, iSH2-kinase communication takes place through the activation loop of kinase and is highly conserved even after nSH2 detachment (Fig. 6).

### Proposed mechanism for p85α inhibition

#### nSH2-activation loop interactions

**Figure 8:**
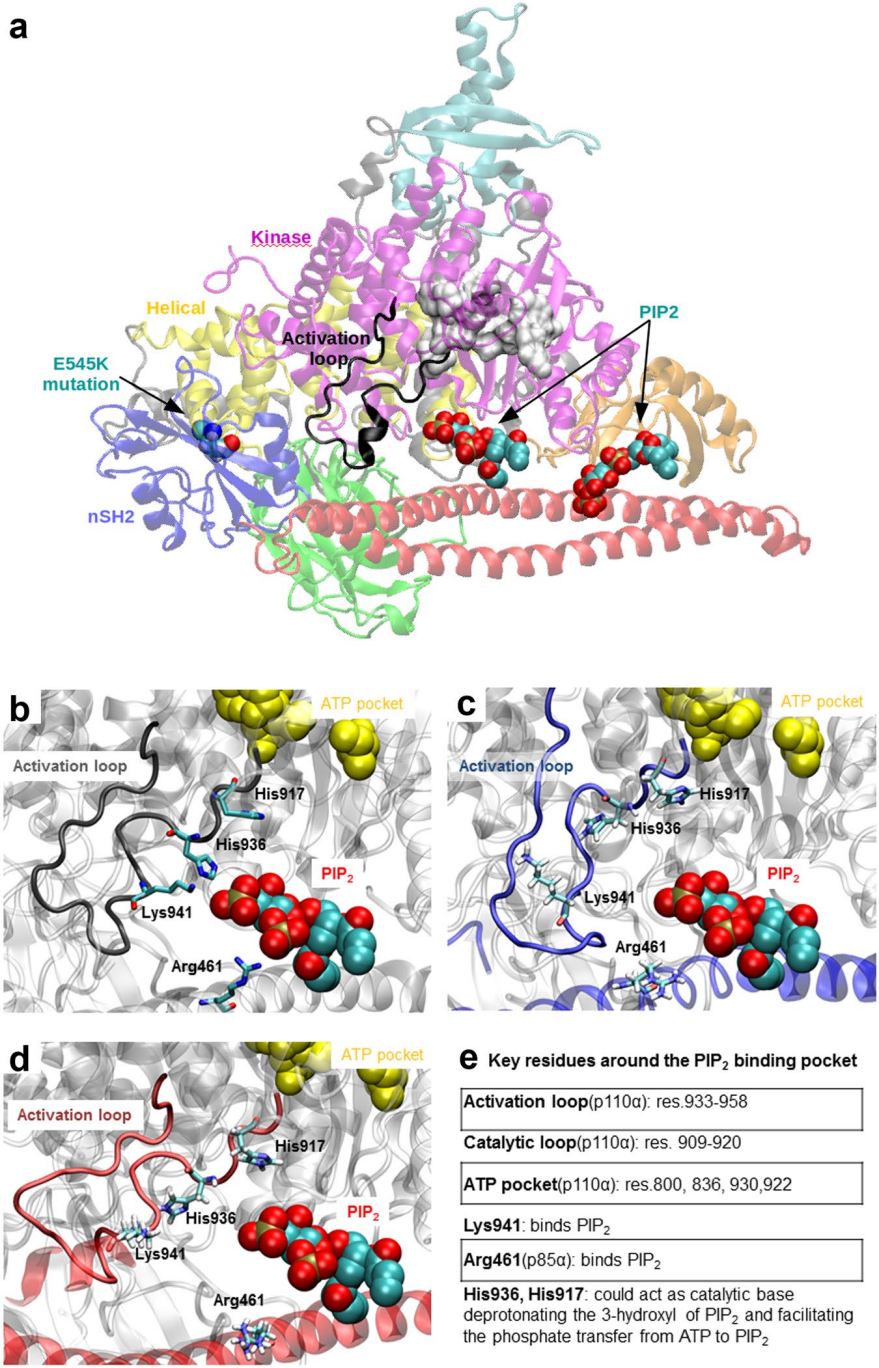
(**a**) Relative position of the activation loop and the PIP2 binding site with respect to the rest of the PI3Ka domains (PDB ID 4OVV). (**b**, **c**, **d**) Snapshots focusing on the area around the PIP2 binding site, taken from the (**b**) WT PI3Kα crystal structure (PDB ID: 4OVV), (**c**) end of the WT run 1 simulation (closed-state) and (**d**) end of the Mutant run1 simulation (open-state). (**e**) Description of key residues and their possible roles in catalysis.

Although the kinase domain of the catalytic region of PI3Ks is highly conserved, members of Class I PI3K exhibit high lipid substrate specificity, determined by residues on the activation loop (res. 933–958) (see Fig. 8a). Amino acid substitutions and structural modelling have shown that the positively charged amino acids of the activation loop are crucial for the selective binding and phosphorylation of phosphatidylinositol (PI) and its phosphorylated derivative phosphatidylinositol 4,5-biphosphate (PIP_2_)^53^. Indeed, a recent crystallographic structure (PDB ID 4OVV) of PI3Kα in complex with a PIP_2_ mimetic, reveals that PIP_2_ binds very close to the activation loop and forms a salt-bridge or water-mediated hydrogen bond with K941 of the activation loop (Fig. 8b)^15^. Moreover, the activation loop forms a salt-bridge between K948 (kinase) and E342 (nSH2) (Supplementary Table S3). The position of PIP_2_ reveals that the previously suggested base for ATP hydrolysis, H917, is too far away from the lipid substrate. Therefore, another base (possibly H936), which is closer to the lipid substrate, should be able to deprotonate the 3′-hydroxyl of PIP_2_. A schematic representation of the area around the activation loop and the lipid substrate binding pocket is given in Fig. 8b-d. Based on this crystallographic structure (PDB ID 4OVV), Miller and co-workers have proposed a possible mechanism by which nSH2 inhibits the catalytic activity^15^. They suggest that the interactions that nSH2 forms with the activation loop regulate its position and keep the latter in its inactive conformation, away from the lipid substrate binding site. The detachment of nSH2 (p85α) from the helical (p110α) domain should disrupt the interactions between nSH2 and the activation loop, and allow the latter to adopt an active-like conformation, in which it will be positioned in the vicinity of the lipid substrate binding site. We find in our simulations that nSH2 forms persistent hydrogen bonds with the activation loop in the WT PI3Kα protein (Fig. 3). More specifically, the E342(nSH2)-K948(activation loop) hydrogen bond is formed both in WT and mutant proteins, while in the WT protein an additional E341(nSH2)-Y947(activation loop) hydrogen bond is formed. We observe a constant and stable interaction of nSH2 with the activation loop in all MD runs of the WT protein, where nSH2 is in contact with the helical domain (Fig. 3f and Supplementary Table S3). In accordance with theoretical predictions based on structural studies^15,18^, the E342(nSH2)-K948(activation loop) bond in the mutant breaks during nSH2 detachment from the helical domain resulting in a total loss of contact and communication between nSH2 and activation loop. Although the nSH2-activation loop interaction is diminished in the mutant open-state (Mutant run 1), we do not observe any significant conformational change in the activation loop that could be attributed to the detachment of nSH2 (Fig. 8c, d). This is also confirmed by the time series of distances between Cα atoms of key residues of the two domains for all simulated systems (Supplementary Fig. S7). Therefore, the positioning of essential residues for binding and deprotonation of the lipid substrate (PIP_2_) remains unchanged even in the absence of nSH2-activation loop interactions. We offer below an explanation why this may be the case.

#### iSH2-activation loop interactions

The dynamics of the activation loop is not significantly altered after nSH2 detachment (Supplementary Fig. S5, activation loop). The average value of RMSF of the activation loop in the WT (simulations: WT run 1, replica 1) ranges between 0.118 ± 0.039 nm − 0.124 ± 0.031 nm (Supplementary Table S4). The same value becomes 0.107 ± 0.027 nm in the mutant open-state simulation (Supplementary Table S4). The low RMSF value of the mutant open state indicates that the activation loop does not experience a conformational change. We observe that while the interaction with the nSH2 domain is lost (Supplementary Fig. S8a, b), the activation loop enhances its interactions with the third helix of the iSH2 domain (p85α residues 587–598), iα3, especially after the detachment of the nSH2 domain (after 500 ns, Supplementary Fig. S8c, d). In the WT crystal structure^15^, this interface is mediated by hydrophobic contacts between L598 (iSH2, p85α) and F945 (activation loop, p110α) and a hydrogen bond between Q591 (iSH2, p85α) and K948 (activation loop, p110α). Ιn the mutant E545K these interactions are enhanced. iSH2 attracts the activation loop through mainly hydrophobic interactions (iSH2 N595 interacts with activation loop Y947, iSH2 K592 with activation loop Y947, iSH2 W597 with activation loop K944, iSH2 L598 with activation loop K941, iSH2 L594 with activation loop F945, iSH2 Q591 with activation loop F945). One hydrogen bond is formed between iSH2 W597 and the activation loop K944, and one salt bridge is formed between the C-terminal carboxylic acid of iSH2 L598 and the side chain of the activation loop K944. We should note finally, that the activation loop does not become more solvent exposed after nSH2 detachment (Supplementary Fig. S8e, f), in accordance with HDX-MS data, in which the deuterium exchange of the region corresponding to the activation loop remained unaffected upon the E545K mutation compared to the WT^41^.

#### Fast kinetics and intrinsic flexibility of nSH2

The detachment of the nSH2 from the helical domain is observed only in one of the eight mutant simulations, which restricts the statistics of the specific event. However, the total number of nSH2-helical hydrogen bonds in all mutant simulations is lower than in the WT runs (Fig. 2 and Supplementary Fig. S3). In the WT simulations, the total number of nSH2-helical hydrogen bonds is ~6, while in the mutant runs it fluctuates between 2 and 4 hydrogen bonds. Interaction energies between nSH2 and helical domains are plotted in Supplementary Fig. S9. It is clearly seen that the nSH2-helical polar interactions are less favorable in the mutant simulations. Nevertheless, the distance fluctuations maps (Supplementary Fig. S6) do not show any significant change in the communication network compared to the WT runs. We could argue that the less favorable nSH2-helical interactions in the E545K mutant replicate simulations could eventually lead to the disruption of the interface resulting in the detachment of nSH2 from the helical.

Large conformational changes in protein kinases take place over a timescale of microseconds to milliseconds^54^. However, we have been able to observe the rare event of the spontaneous detachment of nSH2 from the helical domain in the E545K mutant, on a much shorter timescale of ~500 ns. The relatively fast kinetics of this conformational change could be associated with the intrinsic disorder of the nSH2 domain. It is not a coincidence that most of the crystal structures of WT PI3Kα lack diffraction data of the nSH2 domain. The first data indicating the structure and positioning of nSH2 in the p110-niSH2 complex were given by the H1047R mutant crystal structure^18^. Also, very recently a specific p110α-p85α niSH2 fusion construct (PDB IDs 4L1B, 4L2Y)^16^ was employed in order to capture the nSH2 domain. The free p110α-p85α niSH2 complex that we are using in this study (PDB ID 4OVU)^15^ has shown a considerable degree of ordering in the nSH2 domain. However, even in this structure, the intrinsic disorder of nSH2 is still evident in the relatively high B factors^15^ of nSH2 and the region of iSH2 in the proximity of nSH2. Expensive Metadynamics calculations are required to increase the sampling and provide valuable information about intermediate structures and energetic barriers. However, these calculations are still beyond the current accessible computational time for such large systems (500 K atoms) to reach convergence and provide accurate insights.

## Discussion

**Figure 9:**
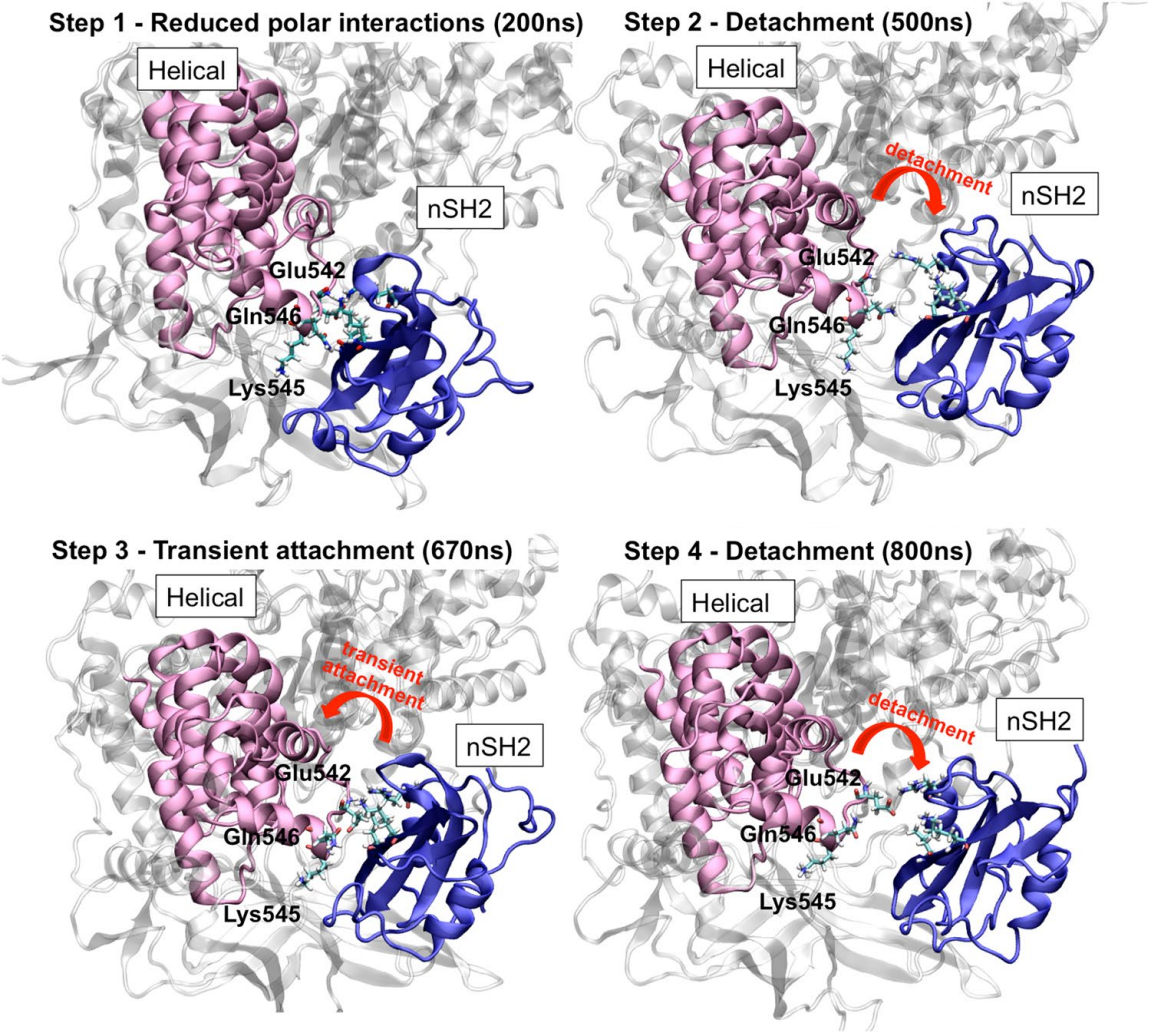
Detachment of the nSH2 (p85α) from the helical (p110α) domain during the simulation. Key hydrogen bonding residues are shown in licorice representation. The helical domain is colored in pink ribbons and the nSH2 domain in blue ribbons.

Biochemical and structural studies suggest that the E545K oncogenic mutant mimics and enhances the physiological activation of the PI3Kα kinase by releasing nSH2 (p85)-helical(p110) inhibitory contacts^21,27,30^. Indeed, our simulations indicate a significant reduction in polar interactions at the nSH2-helical interface in the case of the mutant protein. Interestingly, a complete nSH2 detachment from the helical domain is observed in one out of the eight simulation runs of the mutant. The alteration of the polar contact network around the mutant residue 545K and the breaking of crucial hydrogen bonds between nSH2 and helical domains, lead to the detachment of the two domains that can be observed through a stepwise mechanism (Fig. 9). A critical step towards this significant conformational change is breaking of two specific inter-domain hydrogen bonds (L380(nSH2)-E545(helical) and K382(nSH2)-Q546(helical)) that are present in all PI3Kα complexes (Fig. 2) (Step 1). Subsequently, an interplay between a partially and fully detached nSH2-helical complex is observed where the role of residue E542 (helical) seems to be important (Step 2). The two domains associate transiently again as seen in Fig. 9 (Step 3), but then loss of the R340(nSH2)-E542(helical) critical salt bridge leads to the complete dissociation of the two domains (Fig. 2) (Step 4).

Although the total polar contacts of the nSH2-C2 and nSH2-kinase interfaces remain unaffected, monitoring the distance fluctuations between nSH2 and the helical, C2, and kinase domains revealed that the communication between them is weakened. Moreover, the detachment of the helical-nSH2 domains leads to enhanced flexibility of certain areas of the nSH2, helical and iSH2 domains, in agreement with HDX experiments^30^ (Supplementary Fig. S4).

To further assess the allosteric network of the two proteins, we used the dynamical network analysis method, which indicates that opening of the nSH2-helical interface results in a significant loss of communication between the regulatory and catalytic subunits of the kinase. Moreover, we found that communication signals are transported from the helical to the nSH2 domain through a cluster of helical domain residues R537, P539-E542, T544, and E547.

In the WT protein, the nSH2 domain locks the activation loop in an inactive conformation via a salt-bridge between K948 (p110α) and E342 (p85α). At the same time, K948 is also locked by an interaction with Q591 of the iSH2 domain. The mutation E545K at the helical-nSH2 interface causes a conformational change that breaks the K948 (p110α) – E342 (p85α) interaction with the activation loop, resulting in loss of communication between nSH2 and the activation loop of the catalytic subunit. The loss of contact between the activation loop and nSH2 is accompanied by an enhanced interaction between the activation loop and the iSH2 domain, which maintains an inhibitory role for the activation loop. Loss of contact with iSH2 would also be required for activation; this may be induced by the approach of the high negative charge of PIP2 in the proximity of the positive activation loop (Fig. 8), which is lysine and arginine-rich. We postulate that the release of this inhibitory contact between p85α and the activation loop would allow the activation loop to adopt an active conformation.

We have observed here for the first time in atomic-level detail the spontaneous detachment of the nSH2-helical domains in the E545K mutant and provide a possible mechanism for the loss of regulation that E545K confers based on our simulation results. Insights gained from this study could assist the design of mutant-specific PI3Kα inhibitors that exploit the altered conformation of the mutant with respect to the WT protein.

## Methods

The entire crystal structure of the human WT PI3Kα (PDB ID: 4OVU)^15^ was used for the simulations. A schematic representation of PI3Kα heterodimer with the p110α catalytic and p85α regulatory subunits and all functional domains is depicted in Fig. 1a,b. The E545K mutant was built with a single substitution of the glutamic acid E545 to lysine 545K. Subsequent to introducing the mutation and after minimization (see below), the position of E/K545 backbone nitrogen atom in the helical (p110α) domain remained almost the same allowing the formation of a hydrogen bond with the neighboring L380 backbone oxygen of nSH2 (p85α) (Fig. 1c). All simulations were performed with the GROMACS 5.0.4 software^55^, using the AMBER99SB-ILDN all-atom force field^56^. To decrease the computational cost, the system was solvated in a rhombic dodecahedron box.The TIP3P potential was used for modeling water molecules^57^. The number of the water molecules in the mutant simulations was 110982, while in the WT 110996. The long-range electrostatic interactions were treated using the fast smooth particle-mesh Ewald summation method. The temperature during simulations was kept constant at 310 K using the Nosé-Hoover thermostat with a time constant of 1 ps. The pressure was isotropically maintained at 1 atm using the Parrinello-Rahman coupling with a time constant of 5 ps and compressibility of 4.5e-5 bar^−1^. A time step of 2 fs was used with all bond lengths constrained using the LINCS algorithm. The non-bonded potential energy functions (electrostatic and van der Waals) were smoothly decaying between cut-off distances 0.8–1.0 nm. Prior to MD simulations, both structures were relaxed by 10,000 steps of energy minimization using the steepest descent algorithm, followed by positional restraint equilibration first in the NVT and then in the NPT ensemble for 200 ps each. Finally, unbiased MD simulations were carried out with the atomic coordinates of the systems saved every 2 ps. Two simulations were performed using the WT structure for 1.7 μs in total. In the absence of relevant literature that indicates the protonation state of crucial PI3Kα residues such as 545K, for our initial simulations, we have used the PROPKA algorithm^58,59^ to predict residue protonation states. PROPKA predicted that K379, in the microenvironment of the charged 545K and other positively-charged residues in the vicinity, should be unprotonated, while in the microenvironment of the E545 it should be protonated. We, thus, performed four independent simulations with the mutant residue K379 unprotonated (total aggregation time 3.6 μs) and four with the mutant residue K379 protonated (total aggregation time 7.2 μs).

The trajectory was analyzed using GROMACS v5.0.4^55^ and VMD^51^ tools. To assess the intrinsic flexibility and the apparent plasticity of the protein, maps of distance fluctuations were constructed following the procedure reported by Chiappori *et al*.^46^. In this work, we also use the dynamical network analysis method, which constructs networks of biomolecules constituting nodes (atoms) and edges (connecting atoms), weighted by correlation data. This method has been previously applied to study residue networks of several proteins and protein complexes^43,50,52,60–64^ and explore putative allosteric communication pathways^8^. Using this method, time-dependent positional correlations between residues are calculated. Based on these residue-correlated motions a network representation of the protein is constructed, where the residues (or sets of atoms) are the nodes of the network, which are connected to each other by links (edges) that depend on the node interaction strength.

The method outputs the shortest paths between residues (nodes) as the most dominant mode of their communication. Edges that most frequently belong in short paths are named *“critical edges”* and the nodes connected by these edges are established as *“critical nodes”* for allosteric signal transduction. The last 200 ns of all reported simulations were used for the dynamical network analysis.

All methods are presented in detail in the Supplementary Information.

## Electronic supplementary material

Supporting Information

## Data Availability

All data (input files, output files, trajectories) have been deposited and can be freely accessed at https://repo.vi-seem.eu/handle/21.15102/VISEEM-254.
