# Supplementary material for: Insights into the mechanism of the PIK3CA E545K activating mutation using MD simulations
Source: Sci Rep. 2018 Oct 19;8:15544. doi: 10.1038/s41598-018-27044-6 (PMC6195558; doi:10.1038/s41598-018-27044-6)
Supplement: Supplementary file 1 — Supporting Information [file 41598_2018_27044_MOESM1_ESM.pdf]

Supporting Information

Insights into the mechanism of the *PIK3CA* E545K activating mutation using MD simulations

Hari Leontiadou[1], Ioannis Galdadas[1], Christina Athanasiou[1] and Zoe Cournia[1]*

[1]Biomedical Research Foundation, Academy of Athens, 4 Soranou Ephessiou, 11527, Athens, Greece

*corresponding author

Email: zcournia@bioacademy.gr

Tel: +30 210 6597195

Fax: +30 210 6597545

Specific contacts between E545 – nSH2 and role of the charge reversal

The specific contacts of E545 with the nSH2 domain are:

- K379-E545 (side chain)
- L380-E545 (backbone) as also described in the literature (Adv Biol Regul. 2013 Jan; 53(1): 97–110)

These contacts are lost in the open state of simulation mutant run 1. The K379-E545 hydrogen bond cannot be formed when the 545 is a Lys (due to the charge reversal) and also the L380-E545 is weakened or non-existing in 5 out of 8 mutant simulations (Supplementary Table S4).

The charge reversal plays the following role in the sequence of events present in the detachment:

1) Residues 545K and K379 interact through side chains with van der Waals and hydrogen bond interactions.
2) Gradually they come apart (due to charge repelling) and 545K forms a new salt bridge with D549, while K379 forms a salt bridge with D421 (nSH2).
3) Then 545K interacts with D421 and K379 with K548.
4) Then a hydrogen bond is formed between K379 with the backbone O of L380.

When the side chain hydrogen bond of K379 and 545K is lost, the backbone hydrogen bond between 545K and L380 is gradually lost. The role of the charge reversal is therefore that the neighboring hydrogen bonds are weakened in mutant.

Methods

Model Construction

The atomistic model of the human WT PI3Kα was created based on the recently published crystal structure (PDB ID: 4OVU)[1]. The few missing residues were added and refined through homology and loop modelling using Prime from the Schrödinger suite[2]. The missing residues were: a) in the catalytic domain, p110α: 0-5, 309-318, 410-415, 515-518, 1053-1068 and b) in the regulatory domain, p85α: 322-326, 513-516. A schematic representation of PI3Kα heterodimer with the p110α catalytic and p85α regulatory subunits and all functional domains is depicted in Fig. 1a,b. The E545K mutant was built with a single substitution of residue Glu 545 to Lys. Notice, that after introducing the mutation and after minimization (see below), the position of E/K545 backbone nitrogen atom in the helical (p110α) domain is almost the same allowing hydrogen bond interactions with the neighboring L380 backbone oxygen of nSH2 (p85α) (Fig. 1c).

MD simulations

All simulations were performed with the GROMACS 5.0.4 software[3], using the AMBER99SB-ILDN all-atom force field[4] and the TIP3P water model[5]. As box shape, the rhombic dodecahedron of GROMACS was used, a special case of a triclinic box. This shape was chosen because it truncates the corners of the cubic box and minimizes the water needed for the simulation. The number of the water molecules in the mutant simulations were 110982, while in the WT 110996. The long-range electrostatic interactions were treated using the fast particle-mesh Ewald summation method. The temperature during simulations was kept constant at 310 K using the Nose-Hoover thermostat with a time constant of 1 ps. The pressure was isotropically maintained at 1 atm using the Parrinello-Rahman coupling with a time constant of 5 ps and compressibility of 4.5e-5 bar^{-1}. A time step of 2 fs was used with all bond lengths constrained using the LINCS algorithm. The non-bonded potential energy functions (electrostatic and van der Waals) were smoothly decaying between cut-off distances 0.8 – 1.0 nm. Prior to MD simulations, both structures were relaxed by 10,000 steps of energy minimization using the steepest descent algorithm, followed by positional restraint equilibration first in the NVT and then in the NPT ensemble for 200 ps each. Finally, unbiased MD simulations were carried out with the atomic coordinates of the systems saved every 2 ps (Table S1).

Distance fluctuation (DF) maps

To assess the intrinsic flexibility and the apparent plasticity of the protein, each MD trajectory was projected on the first 10 principal components and maps of distance fluctuations were constructed following the same procedure as in Ref[6]. The distance fluctuation (DF) is defined as the time-dependent mean square fluctuation of the distance r_{ij} between atom i and j, which in our case they correspond to Cα atoms of the residues:

$$DF = \left\langle \left(r_{ij} - \langle r_{ij} \rangle\right)^2 \right\rangle \qquad (1)$$

Where brackets indicate time-average over the trajectory.

Analyses of residues root mean square fluctuation (RMSF), principle component analysis (PCA) and distance fluctuations (DFs) were applied to the last 200 ns of each trajectory, in which the structures were considered to be in equilibrium based on their root mean square deviation (RMSD) from the initial crystal structure (Supplementary Fig. S1). The trajectory was analyzed using GROMACS tools v5.0.4[3] and VMD[7].

Dynamical Network Analysis

Introduction

Dynamical network analysis is a computational method that allows exploring putative allosteric communication pathways.[8] With this method, time-dependent positional correlations between residues are calculated. Based on these residue-correlated motions a network representation of the protein is constructed. Through the dynamical network analysis, the intramolecular interactions in a protein can be collectively represented in the form of a protein structure network, where the residues (or sets of atoms) are the nodes of the network, which are connected to each other by links (edges) that depend on the node interaction strength.

The method outputs shortest paths between residues (nodes) as the most dominant mode of their communication. Edges with the greatest number of shortest paths that cross that edge are named *"critical edges"* and the nodes connected by these edges are established as *"critical nodes"* for allosteric signal transduction.

In our study, the dynamical network analysis was carried out using a similar protocol to Ref[8], which has been previously applied to study a tRNA-protein, as well as several proteins and protein complexes[8-15]. To create a network model for our protein, the nodes and edges need to be created. As mentioned before, in dynamical network analysis, a node represents some set of atoms, e.g. an amino acid within a protein; here, as nodes we have used the Cα atoms of each PI3Kα residue. To define which pairs of nodes will be connected through edges, we have connected nodes, whose residues are within a cutoff distance of 4.5 Å of another residue for at least 75% of the MD simulation. Namely, if residues were within 4.5 Å of another residue for 75% of the trajectory, a correlation value (which will be explained later) was assigned to their edge; otherwise, the correlation value was set to zero. This contact-map filtering prevents the introduction of edges between residues that are physically far apart.

Correlations, edge weights, critical edges, critical nodes

The pairwise correlations are defined as C_{ij},

$$C_{ij} = \frac{\langle \Delta\vec{r_i}(t) \times \Delta\vec{r_j}(t) \rangle}{\left(\langle \Delta\vec{r_i}(t)^2 \rangle \langle \Delta\vec{r_j}(t)^2 \rangle\right)^{1/2}}$$

with $\Delta\vec{r_i}(t) = \vec{r_i}(t) - \langle \vec{r_i}(t) \rangle$, where $\vec{r_i}(t)$ is the position of the ith node.

The pairwise correlations, C_{ij}, of the motion between nodes i and j, determine the information transfer between these nodes. In this way, the motion of node i can be used to predict the direction of motion of node j, since they are correlated.

Edge weights, w_{ij}, are derived from the probability of information transfer across an edge:

$$w_{ij} = -log|C_{ij}| \qquad (2)$$

Thus, the dynamical networks are weighted networks in which the weight (w_{ij}, equation (2)) of an edge between nodes i and j is the probability of information transfer across that edge as measured by the correlation values (C_{ij}) between the two nodes.

In this model, we assume that the most likely biologically relevant pathways are those that minimize the network distance. Finding an optimal path is equivalent to minimizing the distance travelled between nodes in the network. The length of such a path, *Dij,* between distant nodes *i* and *j* is the sum of the edge weights between the consecutive nodes (*k*,*l*) along the path:

$$D_{ij} = \sum_{k,l} w_{k.l} \qquad (3)$$

The shortest distance, Dij^0, between all pairs of nodes in the network is the most dominant mode of communication between the nodes, calculated by using the Floyd–Warshall algorithm.

Edges with the greatest number of shortest paths that cross that edge are named *“critical edges”* and the nodes connected by these edges are established as *“critical nodes”* for allosteric signal transduction.

Community Analysis

The physical network of nodes and edges can be clustered into substructures or communities of nodes that are more densely interconnected to each other than to other nodes in the network. Nodes (residues) that show a high correlation based on their w_{ij} (see equation (2) in SI for its definition), can be grouped in protein regions; these regions are termed *communities*. The community structure is identified by using the Girvan–Newman algorithm, which uses a top-down approach to iteratively remove the edge with the highest betweenness and recalculate the betweenness of all remaining edges until none of the edges remains. The betweenness of an edge, defined as the number of shortest paths that pass through the edge in the network, is used to measure the importance of the edge for communication within the network. According to our simulations, these communities largely reflect the functional domains of PI3KA. Allosteric signalling between communities occurs through particular node pairs that are important for the community crosstalk. These “critical nodes” transfer a relatively large degree of information across their edge. As critical node pairs carry out intercommunity crosstalk, they may provide favorable sites of signal disruption.

To identify residues that are important in transmitting the signal from one region of a protein to another, one should define the path through which the signal is transmitted. According to del Sol et al. (2006), residues that are key in preserving short paths in network communication between the source of the allosteric signal and the end-point of the allosteric signal, are crucial for the transmission of the signal.[17] The shortest (optimal) path between two nodes in a network is determined by the Floyd-Warshall algorithm.[18,19]

The shortest paths between pairs of nodes belonging to two different communities are calculated and analyzed for communication across communities in the network. Of these intercommunity links, all edges connecting any two of these communities are identified. Edges with the greatest betweenness are pinpointed, and the nodes connected by these edges are established as critical for allosteric signal transduction.

The strength of the allosteric signal (A) is defined in this work as indirectly proportional to the sum of the shortest distances from the two nodes, D_{ij}

$$A = \frac{1}{\sum_\iota D_{ij}^0} \qquad (4)$$

This value can be used to compare the strength of the allosteric signal between the wild-type enzyme in different states and/or modifications of the network.

In our calculations, these analyses have been applied for the last 200 ns of each simulation run.

Supplementary Figures

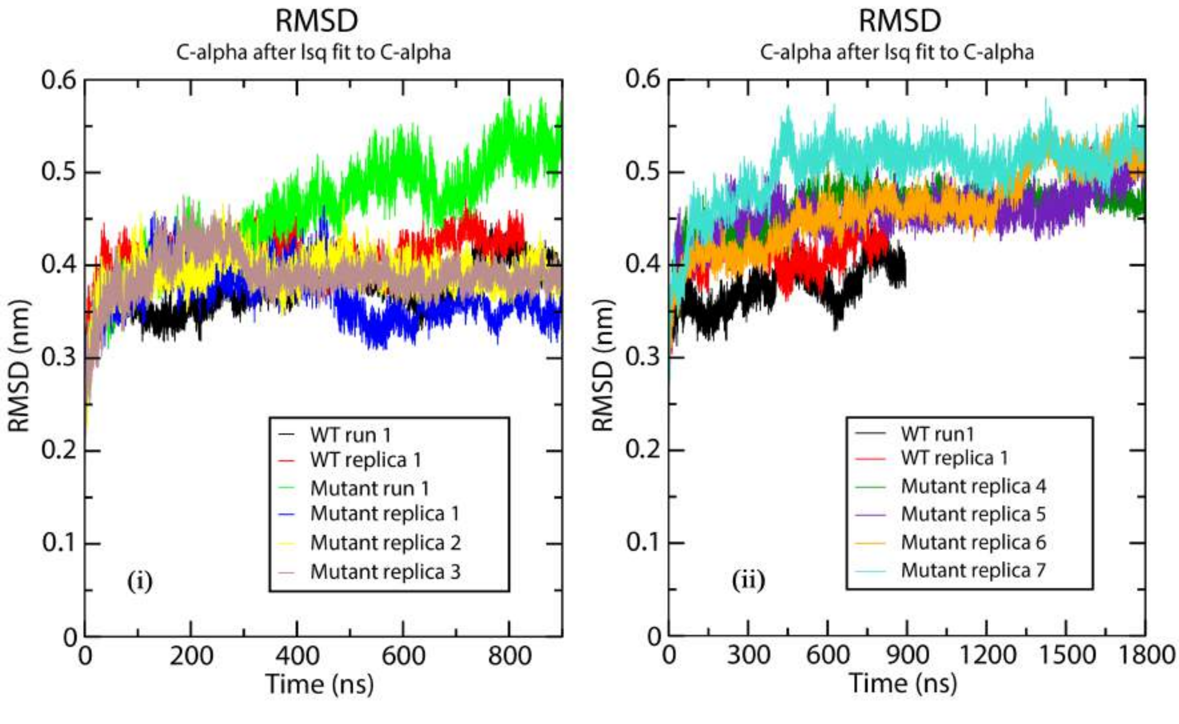

Figure S1. Root mean square deviation from the initial structure as a function of time for all simulation runs of the WT, and E545K mutant protein, where (i) the side chain of K379 is not charged, and (ii) the side chain of K379 is charged.

.

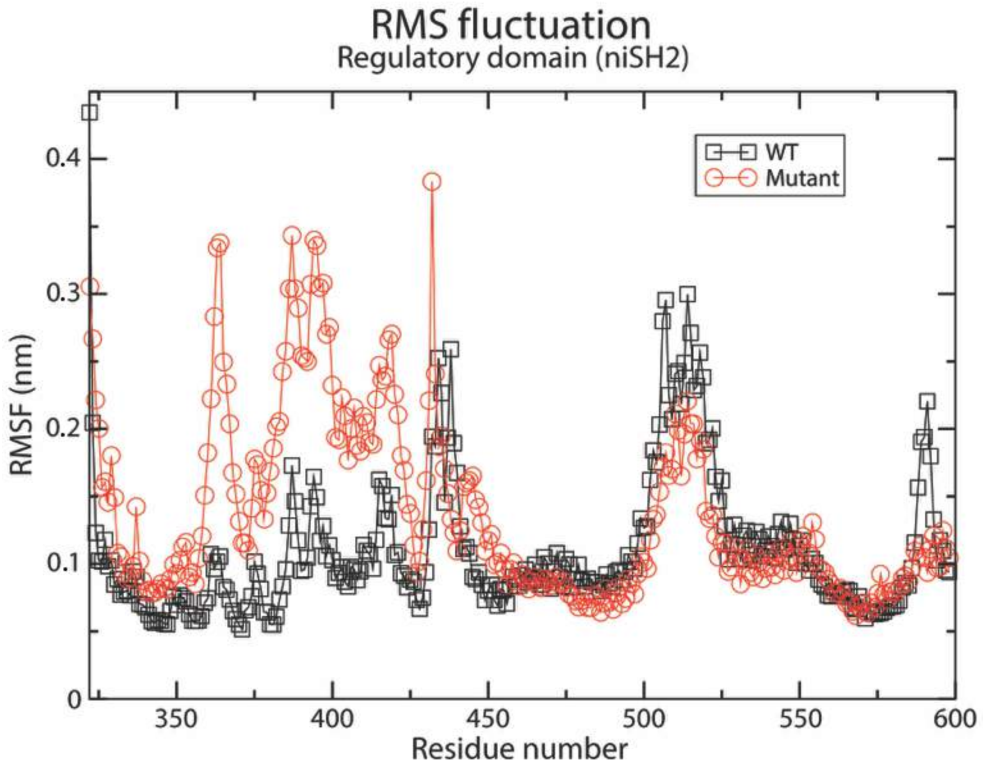

Figure S2. RMSF of niSH2 residues of the regulatory domain of the WT (black) and mutant (red) protein, in which the nSH2-Helical detachment takes place. Significantly increased fluctuations are observed for the residues 322-430 of nSH2 that contact the helical catalytic subunit in the mutant protein.

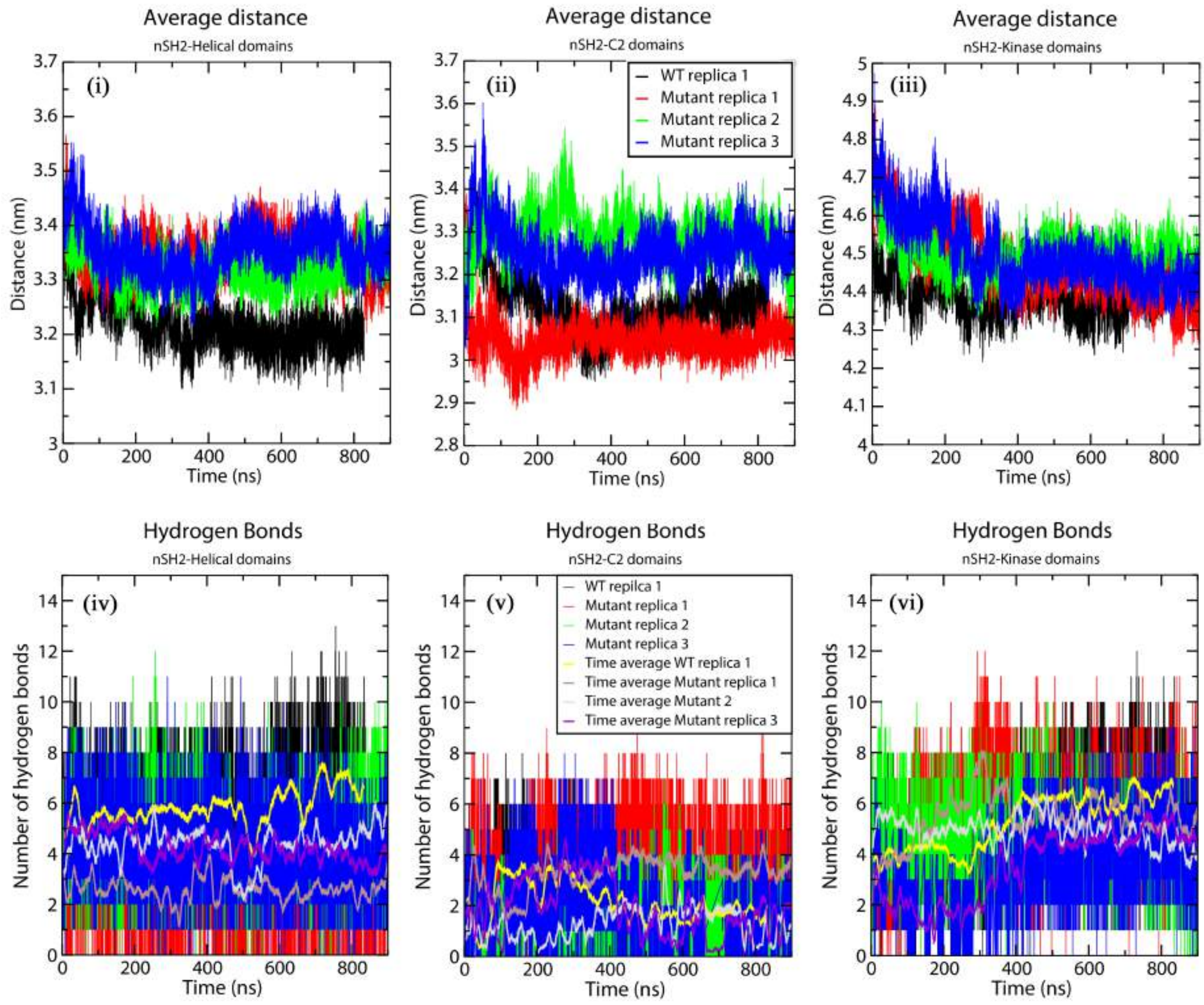
Average distance
nSH2-Helical domains
(i)
Distance (nm)
Time (ns)
Average distance
nSH2-C2 domains
(ii)
WT replica 1
Mutant replica 1
Mutant replica 2
Mutant replica 3
Distance (nm)
Time (ns)
Average distance
nSH2-Kinase domains
(iii)
Distance (nm)
Time (ns)
Hydrogen Bonds
nSH2-Helical domains
(iv)
Number of hydrogen bonds
Time (ns)
Hydrogen Bonds
nSH2-C2 domains
(v)
WT repilca 1
Mutant replica 1
Mutant replica 2
Mutant replica 3
Time average WT replica 1
Time average Mutant replica 1
Time average Mutant 2
Time average Mutant replica 3
Number of hydrogen bonds
Time (ns)
Hydrogen Bonds
nSH2-Kinase domains
(vi)
Number of hydrogen bonds
Time (ns)

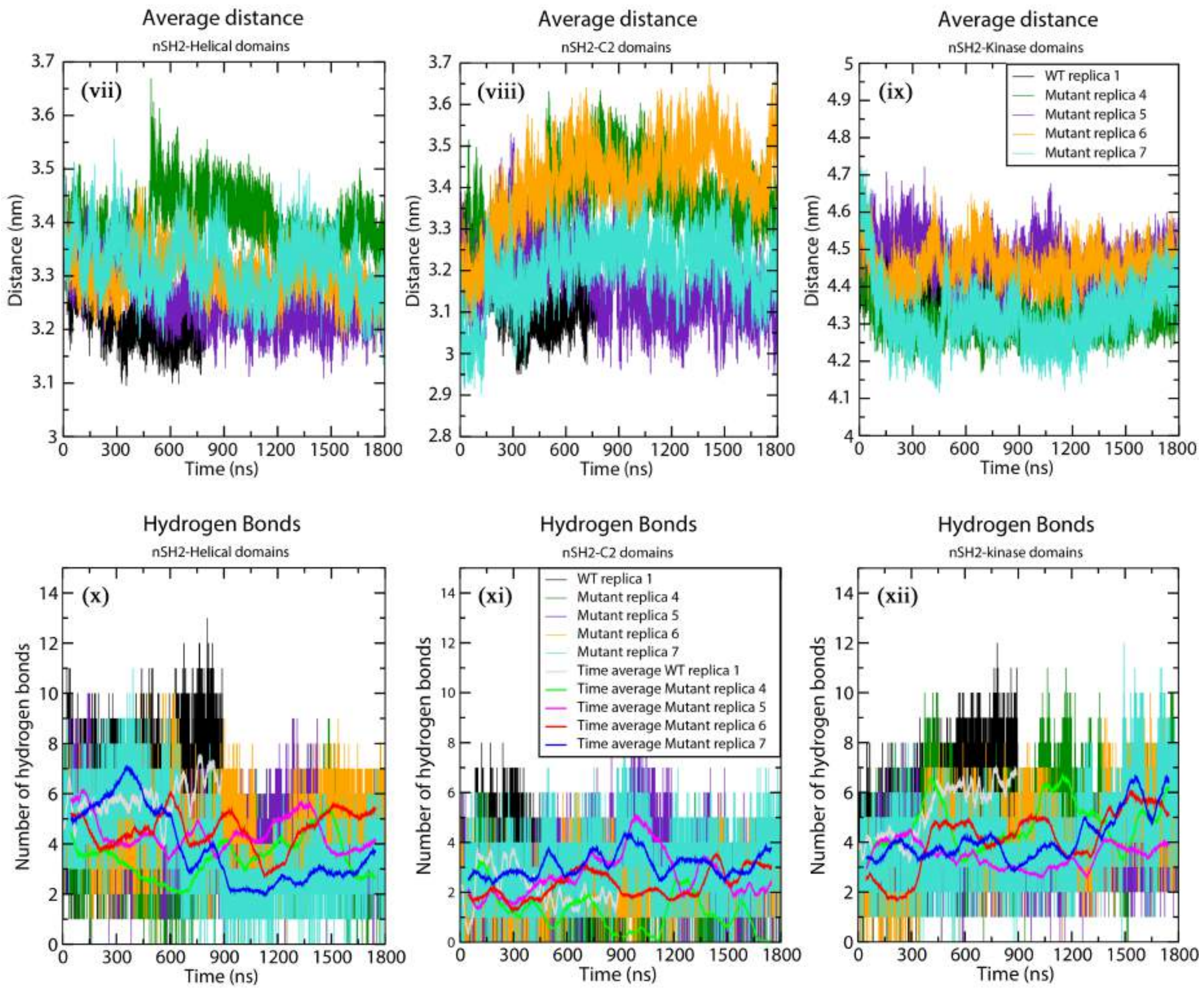

Figure S3. Replicate simulations for the WT (replica 1) and mutant (replica 1-7) proteins. The distance between the centers of mass of the Cα atoms of nSH2-helical (i, vii), nSH2-C2 (ii, viii), and nSH2-Kinase (iii, ix) domains versus time as well as the time dependence of the total number of hydrogen bonds between nSH2-helical (iv, x), nSH2-C2 (v, xi), and nSH2-kinase (vi, xii) domains are presented.

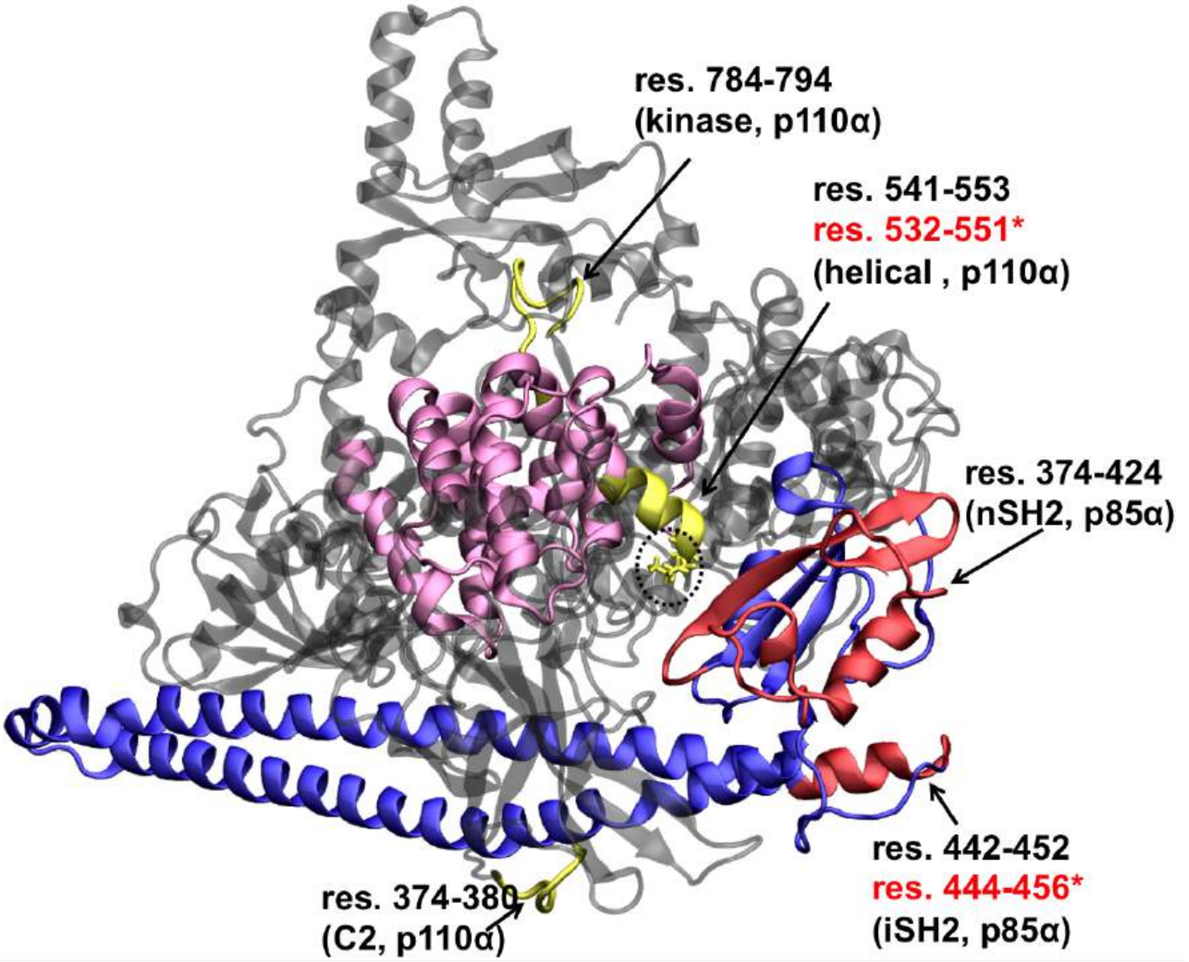

Figure S4. Regions of the detached nSH2 (open-state) of PI3Kα E545K mutant that exhibit enhanced flexibility compared to the simulations of PI3Kα WT (closed-state) as measured with RMSF calculations shown in Supplementary Fig. S5. The E545K mutation site is shown in licorice and indicated in a dotted circle. The p110a subunit is colored grey, with the regions of enhanced flexibility denoted by yellow color and the helical domain by pink. The p85a subunit is shown in blue with the regions of enhanced flexibility denoted by red colo. Asterisks note the corresponding flexible area found from HDX experiments.[20]

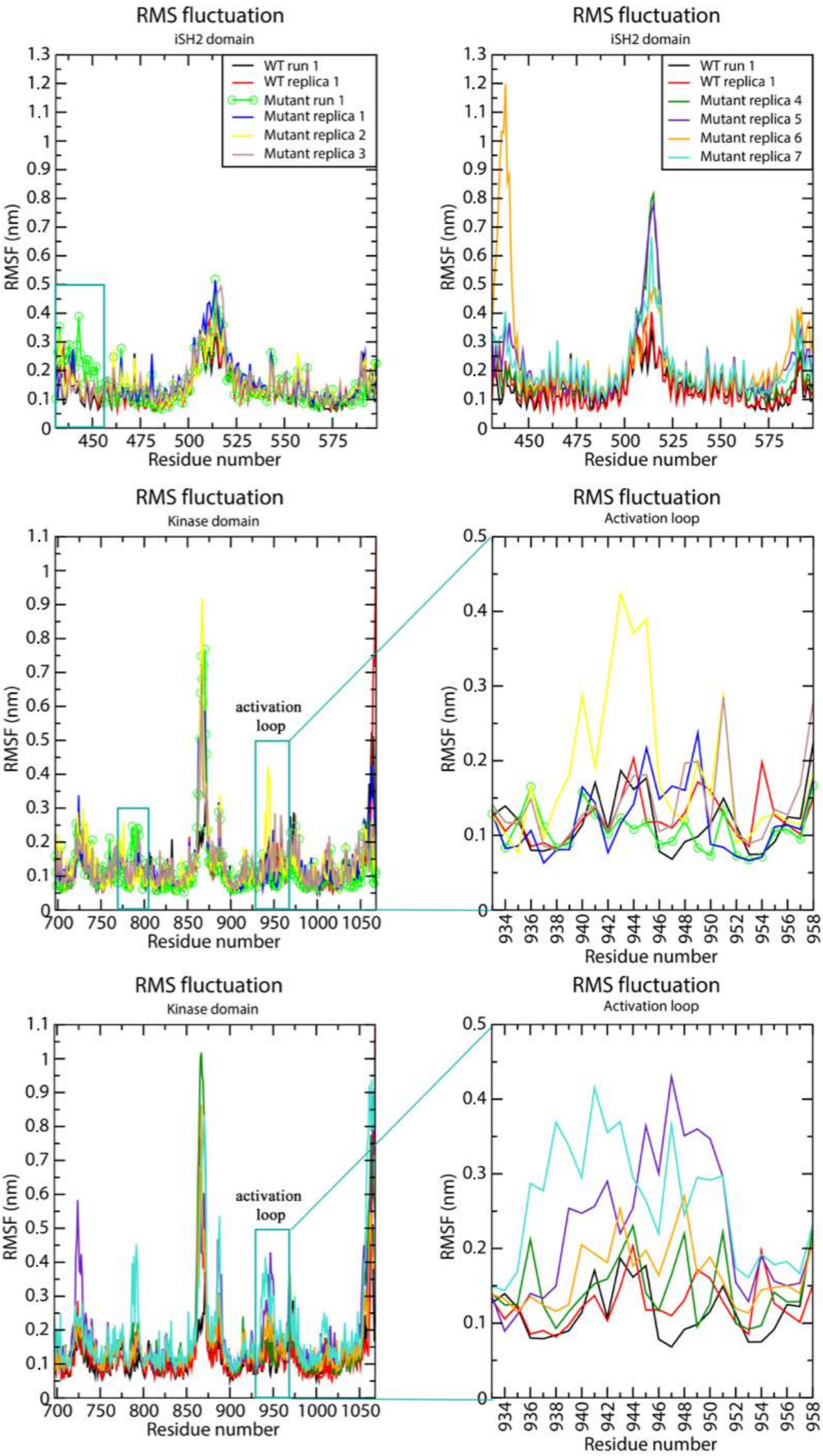
RMS fluctuation
iSH2 domain
WT run 1
WT replica 1
Mutant run 1
Mutant replica 1
Mutant replica 2
Mutant replica 3
RMSF (nm)
Residue number
RMS fluctuation
iSH2 domain
WT run 1
WT replica 1
Mutant replica 4
Mutant replica 5
Mutant replica 6
Mutant replica 7
RMSF (nm)
Residue number
RMS fluctuation
Kinase domain
activation loop
RMSF (nm)
Residue number
RMS fluctuation
Activation loop
RMSF (nm)
Residue number
RMS fluctuation
Kinase domain
activation loop
RMSF (nm)
Residue number
RMS fluctuation
Activation loop
RMSF (nm)
Residue number

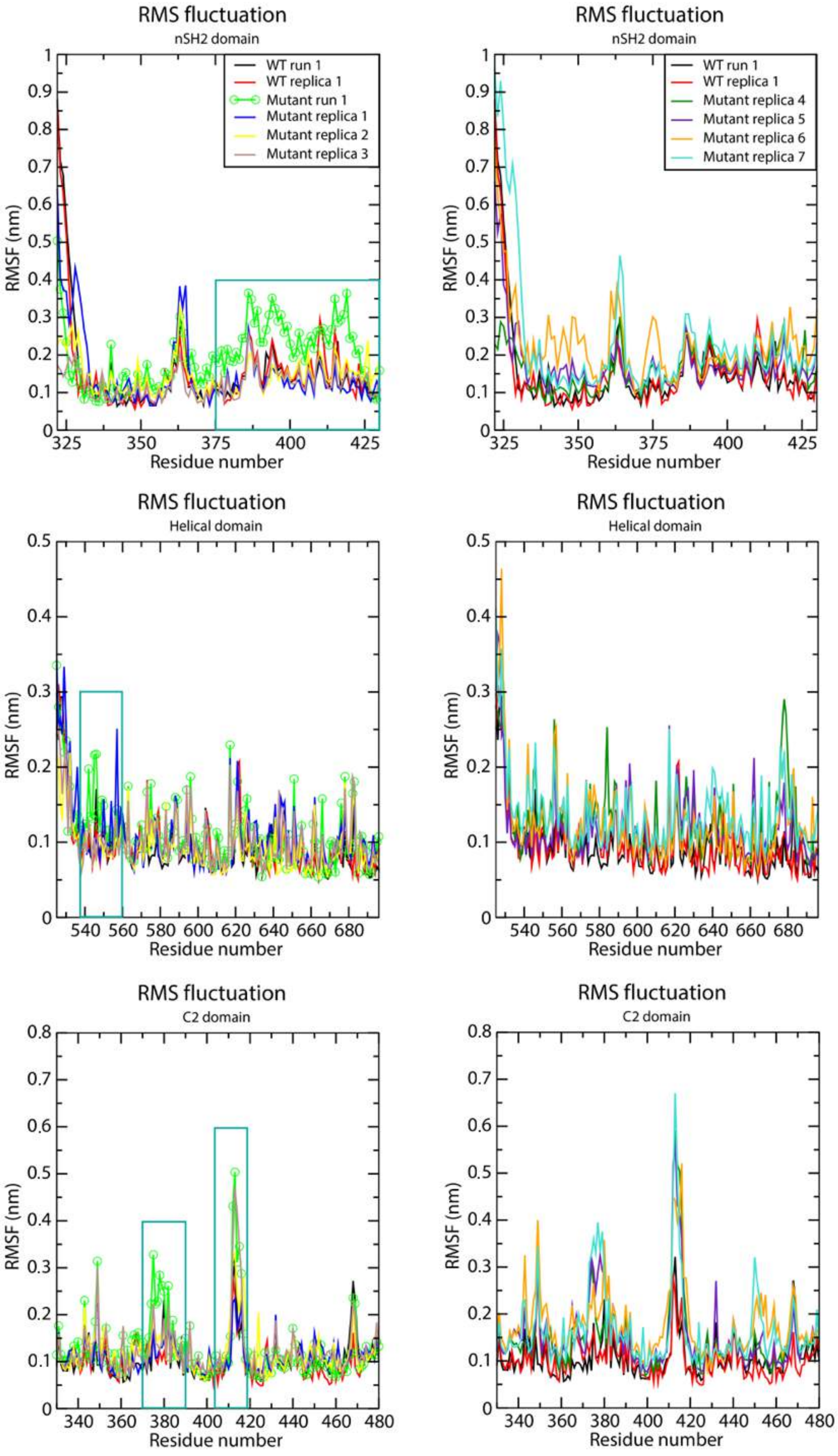
RMS fluctuation
nSH2 domain
WT run 1
WT replica 1
Mutant run 1
Mutant replica 1
Mutant replica 2
Mutant replica 3
RMSF (nm)
Residue number
RMS fluctuation
nSH2 domain
WT run 1
WT replica 1
Mutant replica 4
Mutant replica 5
Mutant replica 6
Mutant replica 7
RMSF (nm)
Residue number
RMS fluctuation
Helical domain
RMSF (nm)
Residue number
RMS fluctuation
Helical domain
RMSF (nm)
Residue number
RMS fluctuation
C2 domain
RMSF (nm)
Residue number
RMS fluctuation
C2 domain
RMSF (nm)
Residue number

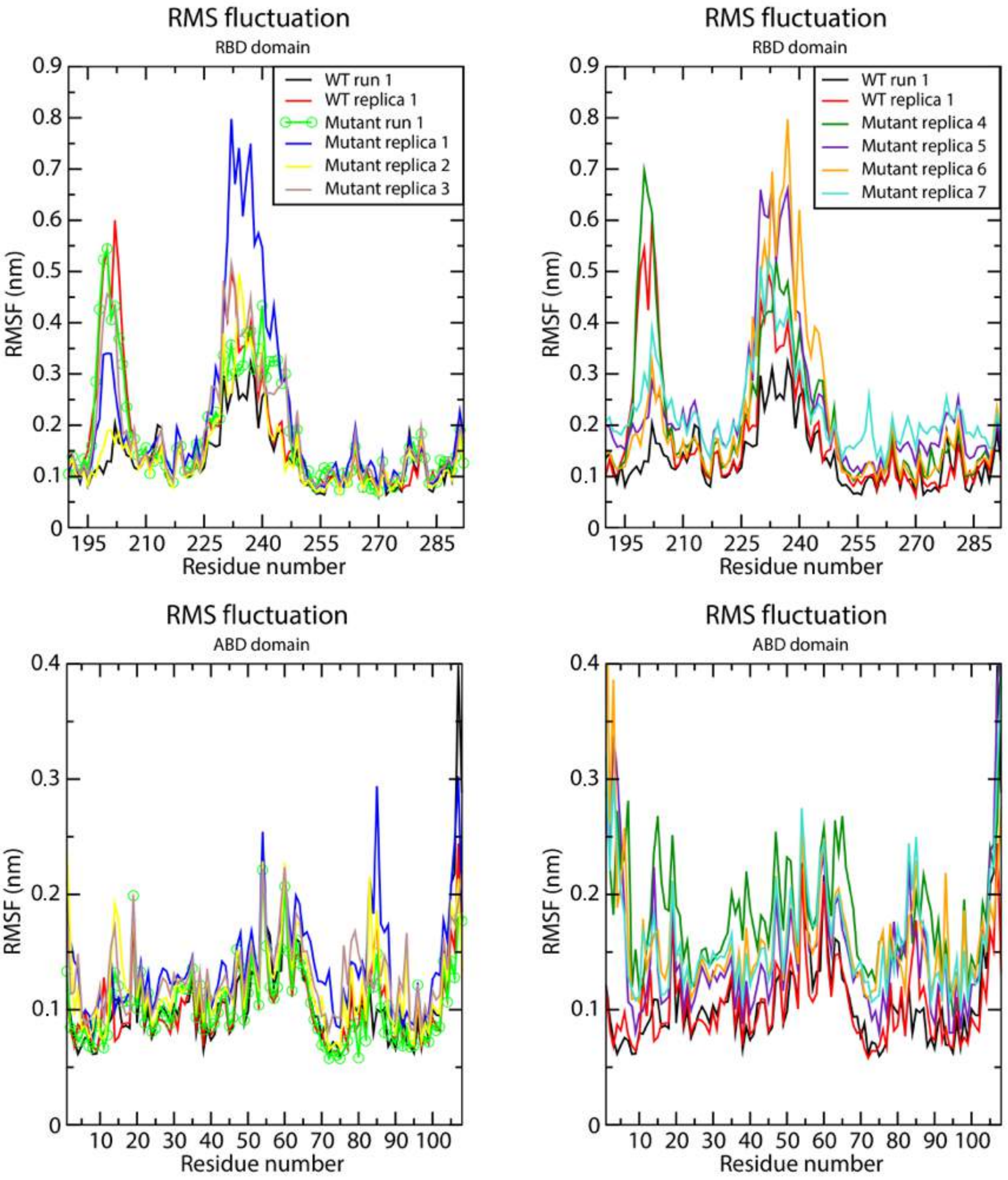

Figure S5. Root mean square fluctuations (RMSF) of residues in functional domains of PI3Kα WT and E545K mutant calculated over the last 200 ns of each simulation. Notice that the mutant run 1 system (green line) is the one where the nSH2 has been detached from the helical domain. Orthogonal shapes inside the graphs include residues of the detached system (mutant run 1) that show enhanced flexibility compared to the rest of the simulations.

nSH2(p85α) - Helical(p110α) distance fluctuations

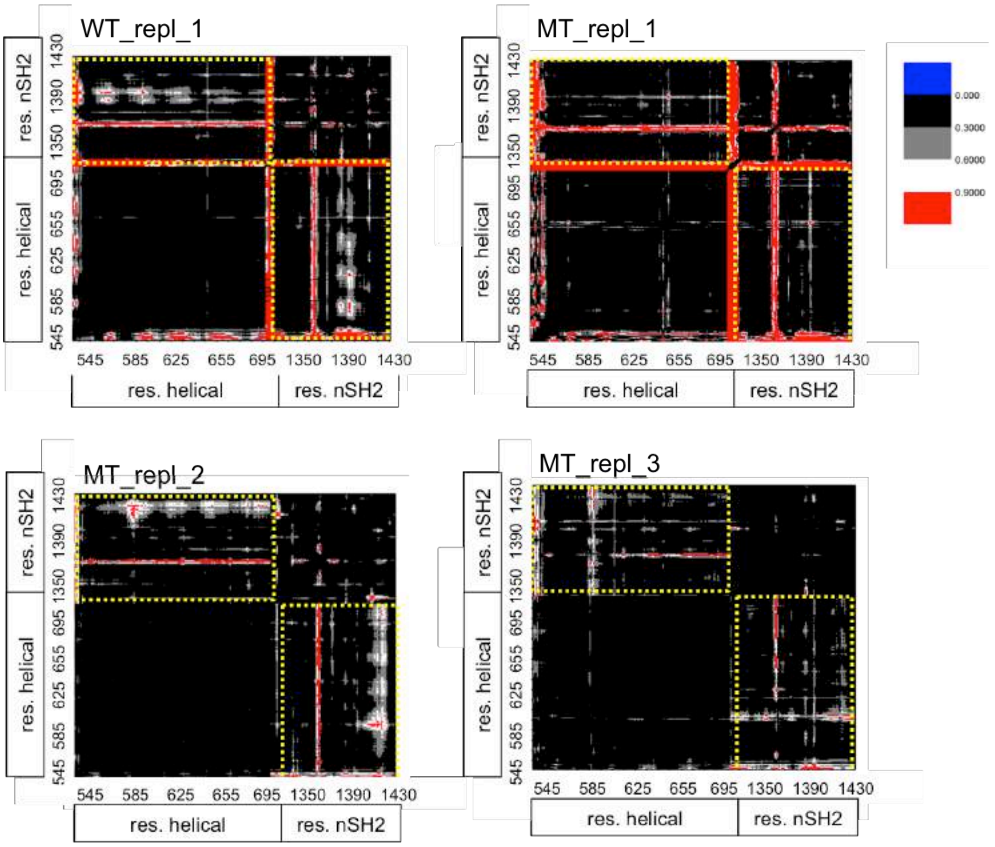

nSH2(p85α) - C2(p110α) distance fluctuations

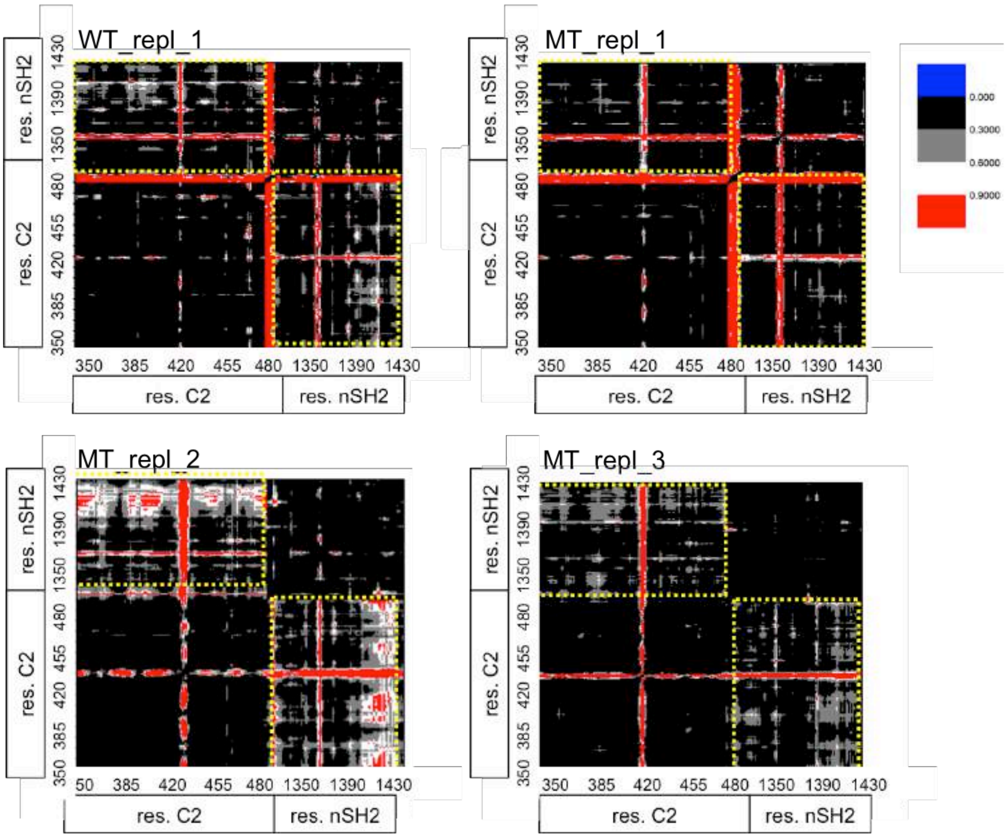

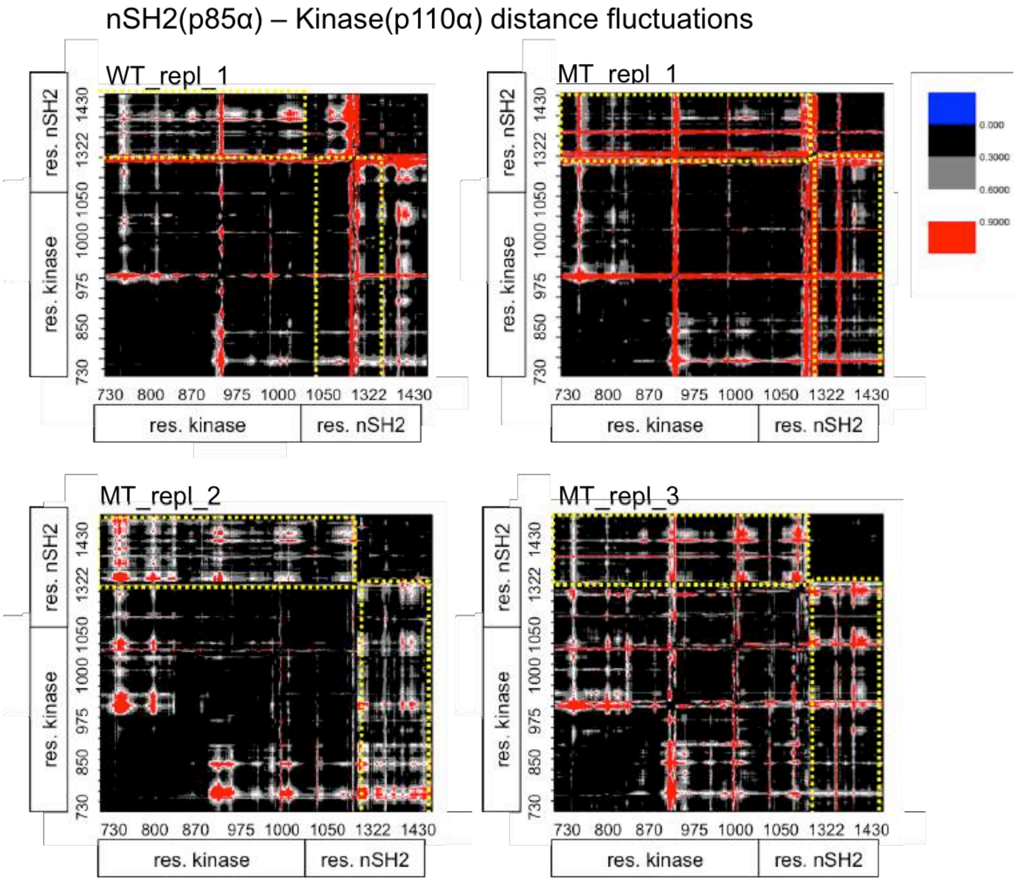

Figure S6. Maps of distance fluctuations (in Å^2) for nSH2-helical, nSH2-C2 and nSH2-kinase domains calculated for all replicate simulation runs. Black areas designate the most rigid parts of the protein while red areas of the graph indicate the strongest conformational variability. Yellow dotted lines include distance fluctuations between residues of the same domain, giving an indication of the inter-domain interactions.

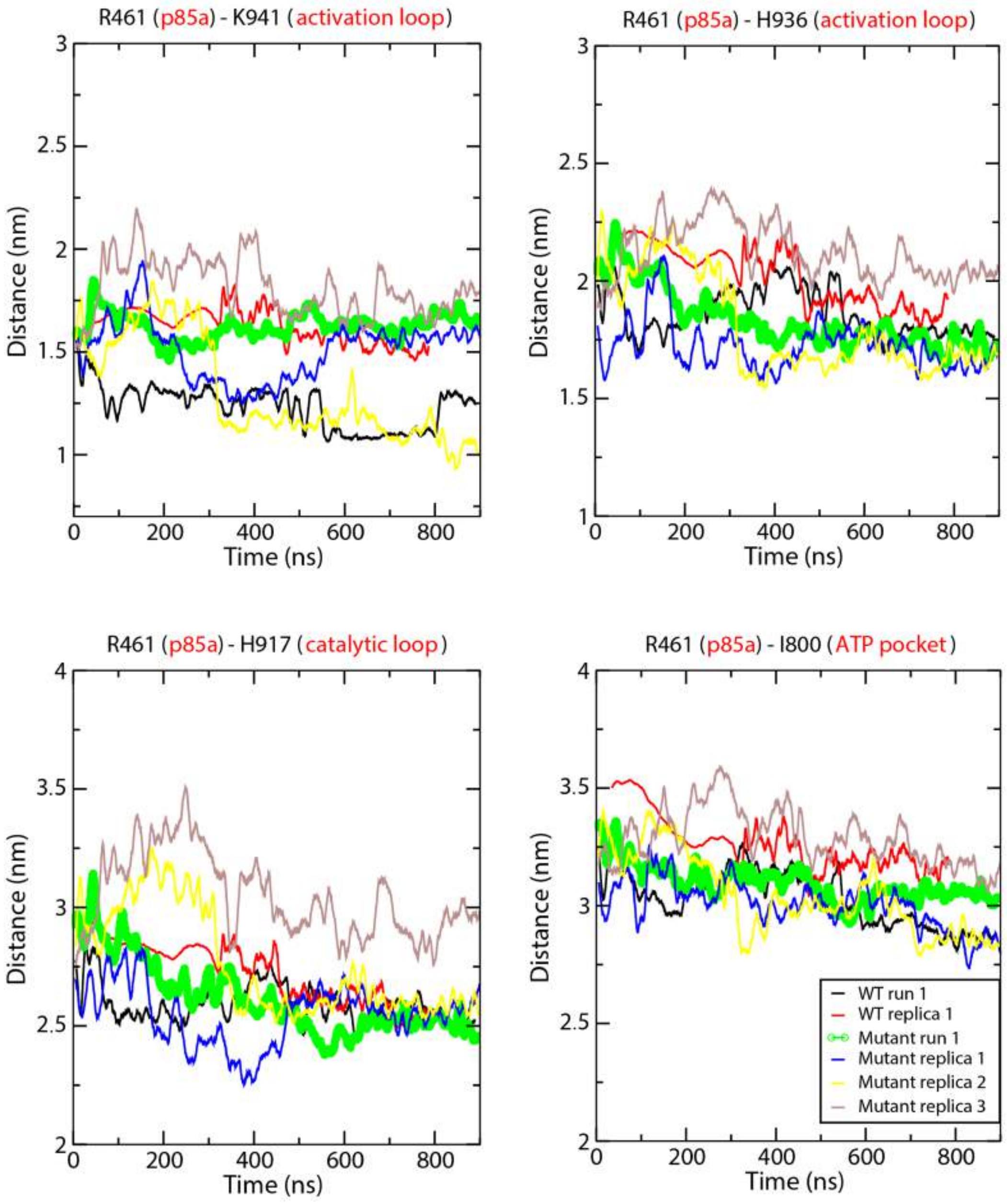

Figure S7. Time series of distances between Cα atoms of key residues around the PIP_2 substrate binding pocket for all simulated systems. All distances were calculated with respect to Cα of R461 (p85α), which is assumed to bind PIP_2 i) K941(activation loop) residue also binding PIP_2 ii, iii) H936(activation loop) and H917(catalytic loop) that could act as a catalytic base and iv) I800, which is part of the ATP pocket.

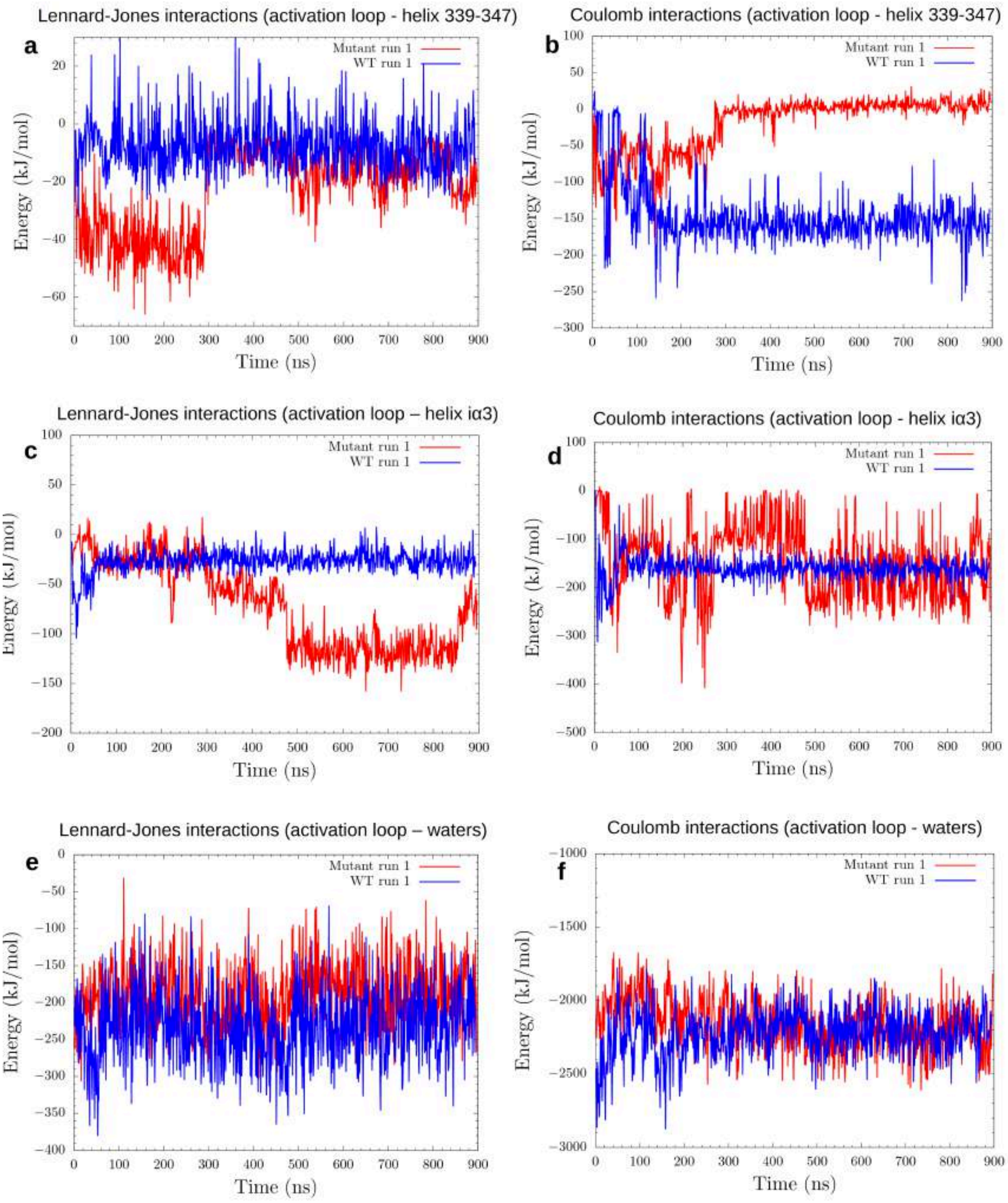

Figure S8. Time series of the (a) Lennard-Jones energies between activation loop and helix 339-347 of nSH2 domain, (b) Coulomb energies between activation loop-helix 339-347 of nSH2 domain, (c) Lennard-Jones energies between activation loop and helix iα3 of iSH2 domain, (d) Coulomb energies between activation loop and helix iα3 of iSH2 domain, (e) Lennard-Jones energies between activation loop and water molecules, (f) Coulomb energies between activation loop and water molecules.

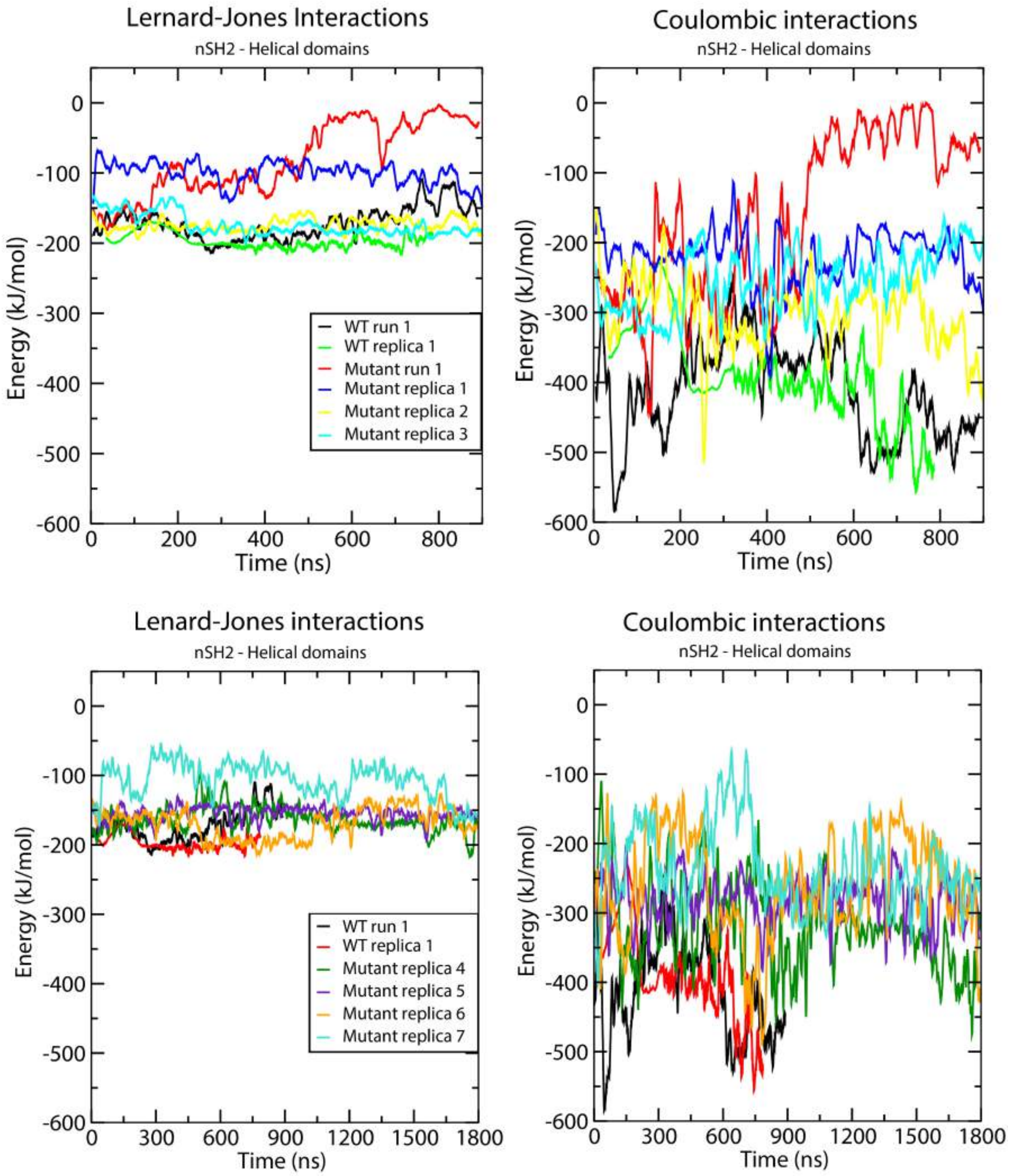

Figure S9. Time series of the Lennard-Jones (left) and Coulombic (right) interaction energies between nSH2 (p85α) and helical (p110α) domains for all simulated systems.

Note: Some of the graphs axes have been manually changed to include a period to indicate the decimal place instead of a comma. Great Britain and the United States are two of the few places in the world that use a period to indicate the decimal place, while many other countries, including Greece, use a comma instead. In our original submission, the decimal place in some plots was indicated with a comma. To follow the submission guidelines, since Scientific Reports uses UK English spelling, we have corrected the format in the graphs and used a period to indicate the decimal place. To do so, we used Adobe Photoshop CS6 software.

Hydrogen Bond and Salt Bridge criteria

VMD was used to calculated hydrogen bonds and salt bridges, and their frequency (percentage of occupancy). A hydrogen bond is defined so that the distance between the donor and acceptor atoms is less than 3.5 Å and the angle between donor-hydrogen atom–acceptor is less than 30 degrees. A salt bridge is considered to be formed if the distance between any of the oxygen atoms of acidic residues and the nitrogen atoms of basic residues are within 4.0 Å, which is defined as the cut-off distance. The frequency of interaction is counted as a percentage of occurrence over all the frames of the trajectory. This frequency may be greater than 100%, because a given residue-pair may contain more than one hydrogen bond, each of which is counted separately.

Table S1. Simulation of mutant run-1 (detachment, open-state). Important hydrogen bonds and salt bridges between nSH2 (residues in red) and helical, C2, activation loop (residues in black) domains, as they evolve during detachment and transient attachment events. Notice that the total percentage of occupancy of a hydrogen bond is averaged over time periods of 50 ns and is a sum of all hydrogen bonds formed between the specific two residues of interest.

Residues	0-50ns	50-100ns	100-150ns	150-200ns	200-250ns	250-300ns	300-350ns	350-400ns	400-450ns
**nSH2 – helical domains**									
**K379-545K**	12%	8%	4%	-	16%	16%	30%	17%	39%
**L380-545K**	86%	90%	49%	-	-	-	-	-	-
**K382-Q546**	113%	107%	71%	-	-	-	-	-	-
**R340-E542**	98%	62%	74%	76%	88%	82%	80%	74%	50%
**R358-E542**	1%	-	32%	-	-	-	-	-	-
	**450-500ns**	**500-550ns**	**550-600ns**	**600-650ns**	**650-700ns**	**700-750ns**	**750-800ns**	**800-850ns**	**850-900ns**
**K379-545K**	26%	7%	4%	-	3%	2%	2%	-	-
**L380-545K**	-	-	-	-	-	-	-	-	-
**K382-Q546**	-	-	-	-	-	-	-	-	-
**R340-E542**	54%	40%	35%	21%	9%	24%	33%	63%	4%
**R358-E542**	-	-	-	-	-	-	-	-	-
**nSH2 – C2 domains**									
**Residues**	**0-50ns**	**50-100ns**	**100-150ns**	**150-200ns**	**200-250ns**	**250-300ns**	**300-350ns**	**350-400ns**	**400-450ns**
**R348-D454**	145%	143%	149%	147%	133%	162%	163%	162%	166%
**D349-R357**	57%	-	-	-	-	-	68%	-	-
	**450-500ns**	**500-550ns**	**550-600ns**	**600-650ns**	**650-700ns**	**700-750ns**	**750-800ns**	**800-850ns**	**850-900ns**
**R348-D454**	175%	171%	172%	170%	170%	164%	155%	156%	167%
**D349-R357**	-	-	-	-	-	-	-	-	-

nSH2 – activation loop									
**Residues**	**0-50ns**	**50-100ns**	**100-150ns**	**150-200ns**	**200-250ns**	**250-300ns**	**300-350ns**	**350-400ns**	**400-450ns**
**E342-K948**	67%	56%	91%	76%	86%	53%	-	-	-
**D337-K948**	44%	41%	76%	74%	45%	53%	78%	79%	81%
**Q345-G946**	73%	62%	35%	16%	15%	13%	-	-	-
	**450-500ns**	**500-550ns**	**550-600ns**	**600-650ns**	**650-700ns**	**700-750ns**	**750-800ns**	**800-850ns**	**850-900ns**
**E342-K948**	-	-	-	-	-	-	-	-	-
**D337-K948**	43%	-	-	-	-	-	-	-	-
**Q345-G946**	-	-	-	-	-	-	-	-	-

Table S2. Simulation of WT run-1 (closed-state). Important hydrogen bonds and salt bridges between nSH2 (residues in red) and helical, C2, activation loop (residues in black) domains in the protein closed-state. Notice that the total percentage of occupancy of a hydrogen bond is averaged over time periods of 50 ns and is a sum of all hydrogen bonds formed between the specific two residues of interest.

**Residues**	**0-50ns**	**50-100ns**	**100-150ns**	**150-200ns**	**200-250ns**	**250-300ns**	**300-350ns**	**350-400ns**	**400-450ns**
**nSH2 – helical domains**									
**K379-E545**	34%	6%	11%	10%	9%	6%	4%	9%	2%
**L380-E545**	90%	112%	113%	99%	97%	101%	94%	101%	103%
**K382-Q546**	137%	93%	123%	99%	173%	182%	183%	168%	138%
**E542-R340**	80%	6%	33%	-	-	36%	13%	42%	-
**R358-E542**	25%	152%	138%	162%	155%	139%	100%	89%	164%
	**450-500ns**	**500-550ns**	**550-600ns**	**600-650ns**	**650-700ns**	**700-750ns**	**750-800ns**	**800-850ns**	**850-900ns**

**K379-E545**	17%	6%	5%	3%	7%	6%	17%	56%	71%
**L380-E545**	100%	103%	103%	97%	110%	114%	91%	59%	73%
**K382-Q546**	164%	178%	177%	81%	82%	75%	5%	-	2%
**E542-R340**	1%	1%	-	1%	3%	-	-	-	-
**R358-E542**	105%	165%	143%	165%	163%	159%	153%	141%	152%
**nSH2 – C2 domains**									
**Residues**	**0-50ns**	**50-100ns**	**100-150ns**	**150-200ns**	**200-250ns**	**250-300ns**	**300-350ns**	**350-400ns**	**400-450ns**
**R348-D454**	8%	123%	144%	144%	137%	131%	147%	157%	161%
**D349-R357**	23%	145%	148%	162%	126%	150%	102%	112%	99%
	**450-500ns**	**500-550ns**	**550-600ns**	**600-650ns**	**650-700ns**	**700-750ns**	**750-800ns**	**800-850ns**	**850-900ns**
**R348-D454**	169%	166%	170%	174%	168%	172%	170%	169%	172%
**D349-R357**	108%	106%	115%	109%	114%	116%	129%	109%	120%
**nSH2 – activation loop**									
**Residues**	**0-50ns**	**50-100ns**	**100-150ns**	**150-200ns**	**200-250ns**	**250-300ns**	**300-350ns**	**350-400ns**	**400-450ns**
**E342-K948**	45%	75%	88%	99%	96%	99%	83%	99%	99%
**D337-K948**	58%	57%	16%	5%	15%	9%	5%	5%	3%
**E342-Y947**	-	28%	57%	38%	-	-	-	-	-
**E341-Y947**	-	-	-	44%	89%	94%	93%	88%	87%
	**450-500ns**	**500-550ns**	**550-600ns**	**600-650ns**	**650-700ns**	**700-750ns**	**750-800ns**	**800-850ns**	**850-900ns**
**E342-K948**	99%	97%	99%	98%	99%	98%	97%	99%	99%
**D337-K948**	4%	3%	2%	3%	4%	5%	15%	17%	51%
**E342-Y947**	-	-	-	-	-	-	-	-	-
**E341-Y947**	91%	93%	91%	94%	93%	93%	92%	90%	92%

Table S3. Important hydrogen bonds in replicate simulations. Results are averaged over the last 100 ns of each simulation run. Residue numbers in red are part of the nSH2 domain of the regulatory p85α domain while residue numbers in black belong to the p110α catalytic subunit. Mutant run 1 is the simulation where nSH2 detaches from the helical domain.

Residues	WT run 1	WT rep 1	MT run 1	MT rep 1	MT rep 2	MT rep 3	MT rep 4	MT rep 5	MT rep6	MT rep 7
**nSH2-helical**										
**K379-E/K545**	48%	-	-	-	-	-	-	-	-	-
**L380-E/K545**	65%	84%	-	-	89%	86%	-	8%	93%	10%
**N417-E/K545**	-	-	-	-	16%	-	3%	2%	-	16%
**K382-Q546**	-	125%	-	-	115%	111%	-	30%	108%	30%
**K382-E547**	45%	93%		62%	40%	45%	97%	88%	74%	46%
**F392-Q546**	-	-	-	-	66%	60%	-	37%	84%	-
**R340-E542**	-	170%	60%	167%	86%	29%	-	98%	-	127%
**R358-E542**	146%	143%	-	32%	31%	60%	-	-	73%	-
**N344-E542**	-	-	-	-	42%	-	-	-	-	-
**helical - helical**										
**D549-E/K545**	-	-	31%	-	94%	93%	-	97%	95%	-
**K548-E/K545**	86%	53%	-	-	-	-	-	-	-	-
**R537-E542**	-	98%	-	-	-	-	-	-	-	-
**nSH2 - C2**										
**R348-D454**	171%	-	157%	-	-	14%	-	-	95%	-
**D349-E453**	-	72%	-	-	-	-	-	-	-	-
**D349-R357**	114%	-	-	-	43%	124%	-	-	-	-
**D352-**	-	-	-	165%	-	-	-	-	-	-

**R357**										
**K419-D390**	-	-	-	61%	-	-	-	41%	-	-
**nSH2 - activation loop**										
**E341-Y947**	91%	-	-	-	-	-	-	-	-	-
**E342-K948**	99%	87%	-	55%	96%	80%	4%	-	13%	50%
**E345-K948**	-	38%	-	2%	12%	-	-	-	-	18%
**D337-K948**	36%	-	--	15%	-	14%	-	-	-	-

Table S4. Average values of RMSF and standard deviation of specific parts of PI3Kα calculated over the last 200 ns of each simulation that show the increased flexibility that results from the nSH2 detachment in the mutant protein (mutant run 1, red boxes).

	C2 domain, residues 374-380	helical domain, residues 541-553
**simulation**	**average RMSF (nm)**	**average RMSF (nm)**
WT run 1	0.139 ± 0.048	0.097 ± 0.024
WT replica 1	0.122 ± 0.022	0.096 ± 0.018
Mutant run 1	0.256 ± 0.036	0.146 ± 0.038
Mutant replica 1	0.129 ± 0.021	0.097 ± 0.019
Mutant replica 2	0.157 ± 0.0.15	0.095 ± 0.015
Mutant replica 3	0.159 ± 0.033	0.096 ± 0.016
Mutant replica 4	0.202 ± 0.055	0.133 ± 0.033
Mutant replica 5	0.293 ± 0.023	0.102 ± 0.012
Mutant replica 6	0.235 ± 0.055	0.130 ± 0.032

Mutant replica 7	0.344 ± 0.032	0.155 ± 0.031	
	activation loop, residues 933-958	nSH2 domain, residues 374-424	
**simulation**	**average RMSF (nm)**	**average RMSF (nm)**	
WT run 1	0.118 ± 0.039	0.146 ± 0.040	
WT replica 1	0.124 ± 0.031	0.154± 0.051	
Mutant run 1	0.107 ± 0.027	0.250 ± 0.054	
Mutant replica 1	0.119 ± 0.045	0.131 ± 0.034	
Mutant replica 2	0.183 ± 0.096	0.147 ± 0.032	
Mutant replica 3	0.141 ± 0.052	0.139 ± 0.037	
Mutant replica 4	0.146 ± 0.043	0.183 ± 0.042	
Mutant replica 5	0.226 ± 0.092	0.165 ± 0.034	
Mutant replica 6	0.164 ± 0.042	0.201 ± 0.050	
Mutant replica 7	0.258 ± 0.078	0.198 ± 0.046	
	iSH2 domain, residues 442-452		
**simulation**	**average RMSF (nm)**		
WT run 1	0.106 ± 0.026		
WT replica 1	0.125 ± 0.031		
Mutant run 1	0.235 ± 0.060		
Mutant replica 1	0.137 ± 0.028		
Mutant replica 2	0.134 ± 0.029		
Mutant replica 3	0.128 ± 0.030		
Mutant replica 4	0.145 ± 0.031		
Mutant replica 5	0.221 ± 0.040		
Mutant replica 6	0.218 ± 0.086		
Mutant replica 7	0.186 ± 0.028		

Table S5. Edges between nSH2 and helical domains for five indicative simulation runs.

WT – run1

	Different Communities	Same Community
**L380**	E545	T544, E542, T371, L370
**K382**	M364, E547	F384, Q546, T544, T369, Y368, G366, H365, F384
**R340**	A20, D17, F16	E342, N344
**R358**	-	E542, I388, V343, L356, T369, Y368, L380
**K379**	E545	T371, L372, N377, K374
**E545**	N417, L420, K379, L380,	L543, E547, K548, D549
**Q546**	F550, D549, K548	I381, K382, T544, Y416
**E542**	-	R358, L380, T544
**K419**	-	N417, D421
**D549**	N417, P418, E546	E545, E547
**N417**	E545, D549	K419, Q515, A414
**N344**	D17, D454	R340, E341, E342

WT – replica1

	Different Communities	Same Community
**L380**	-	E545, T544, E542, T369
**K382**	-	E546, E547, T544, M364, H1365, F384
**R340**	-	E542, R358, S541, L540, S339
**R358**	-	E542, R340, F355
**K379**	-	E545, L372, N377
**E545**	K548	L380, K379, I381, D549, L420

**Q546**	F550	F392, K382, I381, T544, D549
**E542**	-	R358, R340, T544, L540, **R537**
**K419**	-	D549, D421, P418, L420
**D549**	W552,	K419, E546, E547
**N417**	-	E545, K419, Q415, A414
**N344**	D17, F1016	L540, R340, N378, L347

MT – run1 (no edge connects the nSH2 with the helical domain)

	**Different Communities**	**Same Community**
**L380**	-	T371, K382, T369, N378
**K382**	-	H365, L380, F392, G366
**R340**	D29	I338, **E342**, V343
**R358**	-	A360, I338, G336, Y334
**K379**	-	N377, I381, L372, T354
**545K**	-	K548, D549, E547, I543
**Q546**	-	D549, F550, T544
**E542**	T544	L540
**K419**	-	D421, N417
**D549**	-	S553, E546, **545K**, W552
**N417**	-	K419, Q415, A414, L420
**N344**	-	E341, R340, N378

MT – replica1

	**Different Communities**	**Same Community**
**L380**	-	T544, E542, T369 , T371
**K382**	-	T544, Y368, F384, F392
**R340**	-	**E542**, S541, R358, L540, R23, E342

**R358**	-	R340, S339, I338, T369
**K379**		L420, L372, L370
**545K**	Y416	D549, E547, K548
**Q546**	F384, T544	D549, F550, K548
**E542**	-	T544, R340, L380, L540
**K419**	N575	D390, L367, C368, D421, N417
**D549**	-	545K, E546, E547, R516, S553
**N417**		K419, Q415, A414
**N344**	D17, A20	E341, R373

MT – replica5

	**Different Communities**	**Same Community**
**L380**	T544	E542, N378, T369, L372
**K382**	**E546**, **E547**, T544	F392, H365, G366, L370, F384
**R340**	S541	**E542**, L540, R358, D29, S339
**R358**	D29	R340, I338, V343, T369, V360, L356
**K379**	-	L420, V344, T371
**545K**	Y416	E547, D549, K548,
**Q546**	**F392**, K382, H365	T544, D549, F550, K548
**E542**	T544	K419, R340, L540
**K419**	-	D421, P418, K374
**D549**	-	545K, Q546, E547, W552, S553
**N417**	-	L420, A414, Q415, L413
**N344**	-	R340, L347, N378, E341, R348

References

1 Miller, M. S. *et al.* Structural basis of nSH2 regulation and lipid binding in PI3Kalpha. *Oncotarget* **5**, 5198-5208 (2014). doi:10.18632/oncotarget.2263

2 Jacobson, M. P. *et al.* A hierarchical approach to all-atom protein loop prediction. *Proteins* **55**, 351-367, doi:10.1002/prot.10613 (2004).

3 Pronk, S. *et al.* GROMACS 4.5: a high-throughput and highly parallel open source molecular simulation toolkit. *Bioinformatics* **29**, 845-854, doi:10.1093/bioinformatics/btt055 (2013).

4 Aliev, A. E. *et al.* Motional timescale predictions by molecular dynamics simulations: case study using proline and hydroxyproline sidechain dynamics. *Proteins* **82**, 195-215, doi:10.1002/prot.24350 (2014).

5 William L. Jorgensen, J. C., Jeffry D. Madura, Roger W. Impey and Michael L. Klein. Comparison of simple potential functions for simulating liquid water *J. Chem. Phys.* **79** (1983). doi: 10.1063/1.445869

6 Chiappori, F., Merelli, I., Colombo, G., Milanesi, L. & Morra, G. Molecular mechanism of allosteric communication in Hsp70 revealed by molecular dynamics simulations. *PLoS Comput Biol* **8**, e1002844, doi:10.1371/journal.pcbi.1002844 (2012).

7 Humphrey W, D. A., Schulten K VMD: visual molecular dynamics. *J Mol Graph* **14**, 33–38, doi:doi: 10.1016/0263-7855(96)00018-5 (1996).

8 Sethi, A., Eargle, J., Black, A. A. & Luthey-Schulten, Z. Dynamical networks in tRNA:protein complexes. *Proc Natl Acad Sci U S A* **106**, 6620-6625, doi:10.1073/pnas.0810961106 (2009).

9 Vanwart, A. T., Eargle, J., Luthey-Schulten, Z. & Amaro, R. E. Exploring residue component contributions to dynamical network models of allostery. *J Chem Theory Comput* **8**, 2949-2961, doi:10.1021/ct300377a (2012).

10 Scarabelli, G. & Grant, B. J. Mapping the structural and dynamical features of kinesin motor domains. *PLoS Comput Biol* **9**, e1003329, doi:10.1371/journal.pcbi.1003329 (2013).

11 Guo, C. & Zhou, H.-X. Unidirectional allostery in the regulatory subunit RIα facilitates efficient deactivation of protein kinase A. *Proceedings of the National Academy of Sciences* **113**, E6776-E6785, doi:10.1073/pnas.1610142113 (2016).

12 Yao, X.-Q. *et al.* Dynamic Coupling and Allosteric Networks in the α Subunit of Heterotrimeric G Proteins. *Journal of Biological Chemistry* **291**, 4742-4753, doi:10.1074/jbc.M115.702605 (2016).

13 Musille, P. M., Kossmann, B. R., Kohn, J. A., Ivanov, I. & Ortlund, E. A. Unexpected Allosteric Network Contributes to LRH-1 Co-regulator Selectivity. *Journal of Biological Chemistry* **291**, 1411-1426, doi:10.1074/jbc.M115.662874 (2016).

14 McClendon, C. L., Kornev, A. P., Gilson, M. K. & Taylor, S. S. Dynamic architecture of a protein kinase. *Proceedings of the National Academy of Sciences* **111**, E4623-E4631, doi:10.1073/pnas.1418402111 (2014).

15 Miao, Y., Nichols, S. E., Gasper, P. M., Metzger, V. T. & McCammon, J. A. Activation and dynamic network of the M2 muscarinic receptor. *Proc Natl Acad Sci U S A* **110**, 10982-10987, doi:10.1073/pnas.1309755110 (2013).

16 Girvan, M. & Newman, M. E. Community structure in social and biological networks. *Proc Natl Acad Sci U S A* **99**, 7821-7826, doi:10.1073/pnas.122653799 (2002).

17 del Sol, A., Fujihashi, H., Amoros, D. & Nussinov, R. Residues crucial for maintaining short paths in network communication mediate signaling in proteins. *Mol Syst Biol* **2**, 2006.0019, doi:10.1038/msb4100063 (2006).

18 Warshall, S. A Theorem on Boolean Matrices. *J. ACM* **9**, 11-12, doi:10.1145/321105.321107 (1962).

19 Floyd, R. W. Algorithm 97: Shortest path. *Commun. ACM* **5**, 345, doi:10.1145/367766.368168 (1962).

20 Burke, J. E., Perisic, O., Masson, G. R., Vadas, O. & Williams, R. L. Oncogenic mutations mimic and enhance dynamic events in the natural activation of phosphoinositide 3-kinase p110alpha (PIK3CA). Proc Natl Acad Sci U S A 109, 15259-15264, doi:10.1073/pnas.1205508109 (2012).
